# Effect of diabetes technologies on the fear of hypoglycaemia among people living with type 1 diabetes: a systematic review and meta-analysis

**DOI:** 10.1016/j.eclinm.2023.102119

**Published:** 2023-08-04

**Authors:** Meryem K. Talbo, Alexandra Katz, Lee Hill, Tricia M. Peters, Jean-François Yale, Anne-Sophie Brazeau

**Affiliations:** a School of Human Nutrition, McGill University, 21111 Lakeshore Road, Ste-Anne-de-Bellevue, Québec H9X 3V9, Canada; b Faculté de Médecine, Université de Montréal, 2900 Edouard Montpetit Blvd, Montréal, Québec H3T 1J4, Canada; c Department of Paediatrics, Research Institute of the McGill University Health Centre, 5252 de Maisonneuve Boulevard W, Montréal, Québec H4A 3S9, Canada; d Centre for Clinical Epidemiology, and Division of Endocrinology, Lady Davis Research Institute, Jewish General Hospital, 3755 Cote Ste Catherine, Montréal, Québec H3T 1E2, Canada; e Division of Endocrinology and Metabolism, Department of Medicine, McGill University Health Centre, 687 Pine Avenue West Montreal, Montréal, Québec H3A 1A1, Canada; f Montréal Diabetes Research Centre, 900, Saint-Denis, Montréal, Québec H2X 0A9, Canada

**Keywords:** Fear of hypoglycaemia, Hypoglycaemia worries, Behaviours, Type 1 diabetes, Hypoglycaemia, Continuous glucose monitoring, Continuous subcutaneous insulin infusion, Automated insulin delivery

## Abstract

**Background:**

Fear of hypoglycaemia (FOH) significantly disrupts the daily management of type 1 diabetes (T1D) and increases the risk of complications. Recent technological advances can improve glucose metrics and reduce hypoglycaemia frequency, yet their impact on FOH is unclear. This systematic review and meta-analysis (SRMA) aimed to synthesize the current literature to understand the impact of diabetes technologies on FOH in T1D.

**Methods:**

In this SRMA, we searched PubMed, Medline, Scopus, and Web of Science from inception up to May 21st, 2023 for studies assessing the effect of using real-time or intermittently scanned continuous glucose monitors (rtCGM or isCGM); insulin pumps (CSII); and their combinations on FOH as the primary outcome, measured using the Hypoglycaemia Fear Survey (HFS; including total, worries [HFS-W], and behaviours [HFS-B] scores), in non-pregnant adults with T1D. Data was extracted by the first and second authors. Results were pooled using a random-effects model based on study design (RCT and non-RCT), with subgroup analysis based on the type of technology, reported change in hypoglycaemia frequency, and duration of use. Risk of bias was evaluated with Cochrane and Joanna Briggs Institute tools. This study is registered with PROSPERO, CRD42021253618.

**Findings:**

A total of 51 studies (n = 8966) were included, 22 of which were RCTs. Studies on rtCGM and CSII reported lower FOH levels with ≥8 weeks of use. Studies on CSII and rtCGM combinations reported lower FOH levels after ≥13 weeks of automated insulin delivery (AID) use or 26 weeks of sensor-augmented pump (SAP) use. The meta-analysis showed an overall lower FOH with technologies, specifically for the HFS-W subscale. The RCT meta-analysis showed lower HFS-W scores with rtCGM use (standard mean difference [95%CI]: −0.14 [−0.23, −0.05], I^2^ = 0%) and AID (−0.17 [−0.33, −0.01], I^2^ = 0%). Results from non-RCT studies show that SAP users (−0.33 [−0.38, −0.27], I^2^ = 0%) and rtCGM users (−0.38 [−0.61, −0.14], I^2^ = 0%) had lower HFS-W.

**Interpretation:**

We found consistent, yet small to moderate, effects supporting that diabetes technologies (specifically rtCGM, SAP, and AID) may reduce hypoglycaemia-related worries in adults with T1D. Current literature, however, has limitations including discrepancies in baseline characteristics and limited, mainly descriptive, statistical analysis. Thus, future studies should assess FOH as a primary outcome, use validated surveys, and appropriate statistical analysis to evaluate the clinical impacts of technology use beyond just glucose metrics.

**Funding:**

10.13039/501100000024Canadian Institutes of Health Research, 10.13039/100008664Juvenile Diabetes Research Foundation Ltd.

**Research in context**

**Evidence before this study**

Discussions with patient partners highlighted the importance of exploring diabetes technologies, especially in how they might impact a person’s experience with hypoglycaemia and its related fears. Thus, we conducted an initial search to explore the literature on fear of hypoglycaemia (FOH) and any existing reports or reviews on how diabetes technologies impact FOH, specifically in type 1 diabetes (T1D). We searched the electronic databases including PubMed, Medline, Scopus, Cochrane Review, and Google Scholar up to September 2020 using the MeSH term and keywords “type 1 diabetes or insulin dependent diabetes mellitus”, “Technology”, “pump or CSII or insulin pump therapy”, “continuous or flash glucose monitor”, “glucose monitoring system”, “hypoglycemia”, “fear of hypoglycemia”, “distress or worry”, “quality of life” combined with the Boolean operators “AND” “OR”. From this search, we identified a qualitative review on the impact of therapeutic advances (including diabetes technologies) on FOH in adults with T1D. In this review, the authors concluded that although diabetes technologies allowed for improved diabetes management and reduced hypoglycaemia, FOH remained a problem. The authors also reported a lack of consistency in the findings of the studies included in their review that reported on the impact of FOH. However no systematic review or meta-analysis was done yet on this topic while considering the latest advances in technologies.

**Added value of this study**

To our knowledge, this is the most complete review assessing the impact of a variety of diabetes technologies on FOH in type 1 diabetes. This is the first meta-analysis of studies exploring the use of different diabetes technologies on FOH (and related worries and behaviours) while accounting for the change in the frequency of hypoglycaemia. This review found pertinent evidence supporting the role of diabetes technologies in reducing FOH when used for at least four weeks. This decrease in FOH with diabetes technology use was independent of the reduction in hypoglycaemia frequency, further confirming a specific benefit for reducing FOH.

**Implications of all the available evidence**

Overall, our findings, provide insights for clinicians, policymakers, and researchers about the additional benefit of using diabetes technology beyond glucose metrics. These findings can be used to advocate for universal access to such technologies for patients who can benefit from them to improve overall patient outcomes and reduce the burden of the condition.

## Introduction

Globally more than nine million people live with type 1 diabetes (T1D), and as the incidence of T1D increases so does its mental and physical burden.^1^ Insulin delivery is a lifesaving therapy for people living with T1D (PWT1D), and personalized intensive insulin therapy can help individuals achieve their glycaemic targets. The American Diabetes Association (ADA) recommends glycated haemoglobin (HbA1c) goal of <7% (53 mmol/mol) without significant hypoglycaemia to avoid chronic micro- and macrovascular complications.^2^ T1D management includes multiple daily insulin injections (MDI) or continuous subcutaneous insulin infusion (CSII) and measurement of blood glucose (BG) using frequent capillary BG (CBG) or newer technologies such as intermittently scanned continuous glucose monitoring (isCGM, with or without alarms) and real-time continuous glucose monitoring (rtCGM).^3^ CSII delivers rapid insulin at mealtimes and continuously throughout the day to provide a subcutaneous basal infusion rate that can be adjusted. Thus, CSII technology allows for more flexibility to modify one’s insulin regimen according to individual insulin requirements (e.g., pregnancy, hormonal changes, illnesses, and physical activity).^4^ Some CSII systems can be combined with rtCGM and an algorithm to form either a sensor-augmented pump (SAP) or automated insulin delivery (AID). SAP (i.e., low-glucose suspend or predictive low-glucose suspend) can suspend insulin administration when glucose levels decrease based on rtCGM readings, while AID can suspend and increase insulin administration based on changes in rtCGM readings.^3^ These technologies allow PWT1D access to detailed information that helps guide glucose management including monitoring of glycaemic changes and adjusting insulin dosing, thus increasing flexibility and treatment personalization and are shown to improve quality of life (QOL).^5^

While intensive insulin therapy has been shown to improve glycaemic management and delay the progression of long-term complications, it also increases the risk of hypoglycaemia.^6^^,^^7^ Specifically, hypoglycaemia which, in its most severe form, can cause loss of consciousness, seizure, or coma.^8^ Due to the unpleasant, frequent, and sometimes serious consequences of hypoglycaemia, more than 50% of PWT1D develop some degree of fear of hypoglycaemia (FOH).^9^

FOH is a disruptive emotion associated with hypoglycaemia symptoms and its consequences, such as possible injury to oneself or others and fear of being judged for possible socially unacceptable behaviour (inhibited motor or cognitive skills and associated social stigma).^10^ FOH is a significant barrier for optimal T1D management^11^ as many PWT1D fear hypoglycaemia more than the long-term complications of diabetes^12^ and tend to maintain higher glucose levels to avoid experiencing its negative effects.^13^ This is often achieved by engaging in disruptive T1D management behaviours such as injecting less insulin than needed, disconnecting CSII, consuming excessive amounts of carbohydrates, and refraining from participating in physical and social activities.^14^^,^^15^ Thus, FOH becomes a key obstacle to intensifying T1D therapy, thereby increasing the challenges of glycaemic management and the risk of diabetes-related complications.^13^^,^^16^

Given the enhanced flexibility for delivering insulin and for monitoring glucose levels provided by the aforementioned technologies and the reported glycaemia benefits of diabetes technologies compared to conventional treatments,^3^ we hypothesize that using diabetes technologies will decrease FOH regardless of their impact on the frequency of hypoglycaemia. Therefore, the objective of this systematic review and meta-analysis (SRMA) was to determine whether the use of diabetes technologies in adults with T1D is associated with lower FOH and if the type of technology (CSII, isCGM, rtCGM, SAP, or AID) used, duration of use, or if co-occurring hypoglycaemia reduction impacts FOH.

## Methods

### Search strategy and selection criteria

This SRMA follows the Preferred Reporting Items for Systematic Reviews and Meta-Analyses (PRISMA) guidelines.^17^ The protocol was registered on PROSPERO in May 2021 (PROSPERO 2021 CRD42021253618).

A search was conducted using four (4) electronic databases PubMed, Medline, Scopus, and Web of Science from database inception up to May 21st, 2023 using a combination of MeSH terms and/or keywords such as “type 1 diabetes”, “insulin-dependent diabetes mellitus”, “technology”, “CSII or insulin pump therapy”, “continuous or flash glucose monitor”, “hypoglycemia”, “fear”, “distress or worry”, “quality of life”, “closed loop” combined with the Boolean operators “AND” “OR”. Studies published in French or English were included. The detailed search strategies for each database are reported in Appendix 1.

Studies were included if they compared the initiation or addition of a new diabetes technology (such as rtCGM, isCGM, or CSII) to conventional methods (MDI or CBG) in non-pregnant adults (≥18 years) with T1D on insulin therapy and if FOH was a measurable outcome assessed using validated tools such as Hypoglycaemia Fear Survey (HFS). To ensure the inclusion of current literature, we included reports published between 2000 and 2023. Studies not meeting these criteria, or those lasting less than seven days, studies including participants who are pregnant, in critical care, post-transplant, aged <18 years, or using intraperitoneal insulin infusion were excluded. Additionally, studies where technology initiation was combined with another intervention, such as a psychological intervention or an online education module, and studies with a lack of comparison group (either lack of control or pre-post analysis) were also excluded.

To quantify FOH in PWT1D, several validated surveys have been developed. Published in 1987, the 27-item HFS was the first to be developed.^18^ Each item in the 10-item behaviour subscale and the 17-item worry subscale was rated on a five-point scale from 1 (never) to 5 (very often).^18^ It was later updated to reflect modern diabetes therapies and was renamed the Hypoglycaemia Fear Survey II (HFS-II) with 33 items.^19^ The HFS-II tallies a total score (HFS-II T) and two subscale scores on a scale of 0 (never) to 4 (almost always): a 15-item behaviour subscale (HFS-II B), to measure behaviours to avoid hypoglycaemia; and an 18-item worry subscale (HFS-II W), to measure the degree of worries.^19^ Both subscales demonstrate strong psychometric properties, high reliability, and validity and are the most widely used.^18^^,^^19^ Newer and shorter surveys, such as the Fear of Hypoglycaemia 15-item scale (FH-15) were developed to specifically identify individuals with FOH who may benefit from psychological interventions.^20^ Another survey, the Diabetes-Specific Quality-of-Life Scale (DSQOLS), was validated to assess the impact of T1D treatment approaches on QOL, including items on FOH.^21^

### Data analysis

The screening, selection, and data extraction of the articles was independently conducted by two reviewers (MKT and AK), data and risk of bias assessment was done independently by three reviewers (MKT, AK, and LH) and a consensus was reached through discussion as needed. If part of an article was unclear or relevant data was missing, reviewers contacted study researchers. After two emails sent with no reply, the study was excluded from the quantitative assessment.

The title, year of publication, author, and abstract were extracted using Endnote (version X9 3.3). Study design, sample size, methodology, participant characteristics (age, diabetes duration, demographics), technology assessed, tool(s) used to assess the level of FOH, summary statistics (e.g., mean HFS-II scores with standard deviations) were extracted and recorded in an adapted Excel spreadsheet (Table 1, Table 2, Table 3). Bias risk assessment for experimental studies was done using Cochrane’s Risk of Bias tool (RoB) for experimental studies. RoB 2.0 was used to assess the risk of bias in randomized controlled trials (RCTs)^22^ and ROBINS-I was used for the non-randomized intervention studies.^23^ The Joanna Briggs Institute Critical Appraisal Tools were used for observational studies depending on the design.^24^

**Table 1:** Studies assessing the impact of glucose monitoring technologies on fear of hypoglycaemia as a measurable outcome among adults with type 1 diabetes.

Study	Location	Study design, sample size, duration of intervention (weeks)	Baseline characteristics: (age, diabetes duration, Female n (%))	Technology assessed	Comparison	Tools used to assess FOH	Baseline FOH score	Results^a^
**Experimental studies:**								
Beck (2010)	United States	RCT, n = 228, 26 weeks	>18 years (Age, diabetes duration, %sex: NA for this specific subgroup)	rtCGM	CBG	HFS	rtCGM vs CBG HFS-T: 37.4 ± 12.8 vs 37.8 ± 14.3 HFS-W: 30.1 ± 18.3 vs 30.6 ± 18.3 HFS-B: 46.9 ± 11.0 vs 47.3 ± 13.1	rtCGM vs CBG HFS-T: 33.3 ± 11.5 vs 36.0 ± 13.6, p = 0.04 HFS-W: 25.3 ± 15.8 vs 27.7 ± 17.3, p = 0.12 HFS-B: 43.8 ± 11.2 vs 46.8 ± 13.3, p = 0.03
Bolinder (2016)	Europe	RCT, n = 239, 26 weeks	isCGM: 42 [33–51] years, 20 [13–27] years, F: 42 (35%) CBG: 45 [33–57] years, 20 [12–32] years, F: 61 (51%)	isCGM	CBG	HFS-II W and HFS-II B	NA	isCGM vs CBG (mean [95%CI]) HFS-W: 14.8 [12.6, 17.1] vs 16.0 [13.7, 18.4], p = 0.42 HFS-B: 13.7 [12.7, 14.8] vs 13.8 [12.6, 14.9] p = 0.98
Heinemann (2018)	Germany	RCT, n = 149, 22–26 weeks	rtCGM: 45.8 ± 12.0 years, 20.9 ± 14.0 years, F: 35 (47%) CBG: 47.3 ± 11.7 years, 21.6 ± 13.9 years, F: 25 (34%)	rtCGM	CBG	HFS-II T, HFS-II W and HFS-II B	rtCGM vs CBG: HFS-T: 53.0 [37.1, 69.8] vs 55.0 [35.0, 65.0] HFS-B: 20.0 [14.0, 27.0] vs 18.5 [14.0, 24.0] HFS-W: 31.0 [23.0, 41.8] vs 36.0 [19.0, 43.0]	rtCGM vs CBG: HFS-T: 37.0 [24.0, 51.0] vs 42.2 [24.5, 59.0], p = 0.08 HFS-B: 14.0 [11.0, 20.8] vs 16.0 [12.0, 24.0], p = 0.21 HFS-W: 24.0 [10.3, 30.8] vs 26.0 [13.0, 39.0], p = 0.34
Ehrmann (2019)	Germany	Secondary analysis of Heinemann (2018) n = 141, 22 weeks	rtCGM: 45.8 ± 12.0 years, 26.1 ± 14.0 years, F: 35 (47%) CBG: 47.3 ± 10.1 years, 20.8 ± 13.1 years, F: 21 (32%)	rtCGM	CBG	HFS-II T, HFS-II W and HFS-II B	rtCGM vs CBG HFS-T: 53.6 ± 21.8 vs 51.5 ± 20.4 HFS-W and HFS-B: NA	rtCGM vs CBG (adj mean ± SE): HFS-T: 39.10 ± 2.10 vs 44.94 ± 2.24, p = 0.04 HFS-B: 16.27 ± 0.89 vs 18.44 ± 0.95, p = 0.06 HFS-W: 22.84 ± 1.48 vs 26.49 ± 1.58, p = 0.09
Lind (2017)	Sweden	Crossover RCT, n = 140, 26 weeks each	44.6 ± 12.7 years, 22.2 ± 11.8 years, F: 62 (44%)	rtCGM	CBG	Swe-HFS-B and Swe-HFS-W	SWE-HFS-B: 1.92 ± 0.58 SWE-HFS-W: 0.85 ± 0.67	rtCGM vs CBG (Least-square means (95% CIs)) SWE-HFS-B: 1.93 (1.83, 2.03) vs 1.91 (1.81, 2.00), p = 0.45 SWE-HFS-W (mean ± SD): 0.80 ± 0.68 vs 0.81 ± 0.67, p = NA
Lind (2021)	Sweden	Prospective follow-up of previous Lind 2017, n = 91, 52–78 weeks	45.7 ± 12.7 years, 24.6 ± 11.9 years, F: 41 (38%)	rtCGM	CBG	Swe-HFS-B and Swe-HFS-W	Swe_HFS_B: 1.91 ± 0.61 Swe_HFS_W: 0.88 ± 0.77	Swe_HFS_B: 1.84 ± 0.60, p = 0.07 Swe_HFS_W: 0.78 ± 0.65, p = 0.02
Little (2014)^b^	United Kingdom	2 × 2 RCT, n = 96, 104 weeks	48.6 ± 12.2 years, 28.9 ± 12.3 years, F: 62 (64%)	rtCGM	CBG	HFS-II T, HFS-II W and HFS-II B	HFS-T: 58 ± 26 HFS-W: 35 ± 17 HFS-B: 24 ± 11	rtCGM vs CBG HFS-T: 45 ± 25 vs 45 ± 24, p = 0.96 HFS-W: 24 ± 17 vs 25 ± 17, p = 0.98 HFS-B: 20 ± 11 vs 21 ± 10, p = 0.94
Oskarsson (2018)	Europe	RCT, n = 163, 26 weeks	isCGM arm: 42 [32–53] years, 19 [14–25] years, F: 25 (31%) CBG arm: 44 [34–53] years, 19 [11–31] years, F: 33 (41%)	isCGM	CBG	HFS-II W and HFS-II B	isCGM vs CBG: HFS-B: 11.9 ± 6.4 vs 12.7 ± 7.3 HFS-W: 15.0 ± 10.1 vs 19.0 ± 14.0	isCGM vs CBG: HFS-B: 13.4 ± 5.6 vs 14.2 ± 7.3 HFS-W: 14.9 ± 11.8 vs 18.4 ± 13.5 Difference in adjusted means in intervention and control (95% CI) HFS-B subscale: −0.3 (−2.0, 1.4), p = 0.76 HFS-W subscale: −1.0 (−4.6, 2.6), p = 0.59
Polonsky (2017)	United States	RCT, n = 155, 24 weeks	48 ± 13 years, 12 ± 12 years, F: 69 (45%)	rtCGM	CBG	HFS-II W	rtCGM vs CBG: 15.8 ± 12.3 vs 17.3 ± 13.2	rtCGM vs CBG: 13.5 ± 10.6 vs 17.7 ± 14.9, p = 0.15
Pratley (2020)	United states	RCT, n = 203, 26 weeks	68 [65, 67], 37 [24, 49], F: 105 (52%)	rtCGM	CBG	HFS-II-W	rtCGM vs CBG: 0.9 [0.4, 1.6] vs 0.9 [0.4,1.5]	rtCGM vs CBG: 0.7 [0.3, 1.2] vs 0.8 [0.5, 1.5], p = 0.22
Miller (2022)	United states	Follow up to Pratley 2020, n = 94, 52 weeks	68 [65, 72], 37 [25, 49], F: 100 (52%)	rtCGM-rtCGM	CBG-rtCGM	HFS-II W	rtCGM-rtCGM: 0.9 [0.4, 1.6] CBG-rtCGM: 1.0 [0.5, 1.5]	At 52 weeks: rtCGM-rtCGM: 0.8 [0.4, 1.3], p = 0.03 CBG-rtCGM: 0.8 [0.4, 1.3], p = 0.50
Reddy (2018)	United Kingdom	Randomize, non-masked parallel group study, n = 40, 8 weeks	49.6 [37.5–63.5] years, 30.0 [21.0–36.5] years, F: 16 (40%)	rtCGM	isCGM	HFS-II T, HFS-II W and HFS-II B	rtCGM vs isCGM HFS-T: 59.5 [37.0–78.0] vs 42.5 [32.0–56.5] HSF-B: 21.0 [13.5–31.0] vs 17.5 [12.5–24.5] HFS-W: 40.5 [24.0–52.5] vs 27.5 [18.0–34.5]	rtCGM vs isCGM HFS-T: 49.5 [28.0–74.0] vs 42.0 [28.5–65.5], p = 0.02 HSF-B: 20.0 [10.5–26.0] vs 15.0 [11.5–25.5], p = 0.36 HFS-W: 30.0 [17.5–44.0] vs 31.0 [15.5–46.0], p = 0.02 p-value for median change from baseline
Reddy (2018)	United Kingdom	Extension (pre-planned), n = 36, 16 weeks	49.5 [37.5–63.5] years, 30.0 [21.0–36.5] years, F: 16 (40%)	rtCGM extended (16wks)	isCGM (8weeks) to rtCGM (8weeks)	HFS-II T, HFS-II B and HFS-II W	Intervention vs control HFS-T: 54.0 [28.0, 78.0] vs 42.0 [27.7, 66.7] HSF-B: 20.0 [11.0, 28.05] vs 15.0 [11.2, 27.7] HFS-W: 32.0 [16.7, 49.7] vs 31.0 [15.2, 47.0]	Intervention vs control HFS-T: 47.0 [29.5, 73.2] vs 38.0 [27.5, 50.5], p = 0.94 HSF-B: 18.5 [10.2, 24.7] vs 15.5 [11.2, 22.7], p = 0.41 HFS-W: 29.5 [18.2, 40.5] vs 21.5 [14.0, 36.5], p = 0.51
Van Beers (2017)	The Netherlands	Crossover RCT, n = 52, 16 weeks each	48.6 ± 11.6 years, 30.5 [18.5–40.8] years, F: 24 (46%)	rtCGM	CBG	HFS-W	rtCGM vs CBG (mean [95%CI]): 38.9 [33.5, 44.2] vs 38.2 [32.8, 43.6]	Mean at end of intervention phase 32.5 vs at end of CBG phase 38.9. Between groups difference [95%CI] HFS-W: 6.4 [1.4–11.4], p = 0.014
Visser (2021)	Belgium	RCT, n = 254, 26 weeks	rtCGM:v42.8 ± 13.8 years, 18 [10–30] years, F: 46 (36%) isCGM: 43.0 ± 14.5 years, 17 [8–28] years, F: 51 (40%)	rtCGM	isCGM	HFS-II W and HFS-II B	rtCGM vs isCGM (mean [95%CI]) HFS-W: 18.7 [16.5, 20.8] vs 18.8 [16.7, 21.0] HFS-B: 19.7 [18.7, 20.7] vs 20.1 [19.1, 21.0]	rtCGM vs isCGM (mean [95%CI]) HFS-II W: 15.4 [13.3, 17.5] vs 18.0 [15.8, 20.1], p = 0.007 HFS-II B: 19.0 [18.0, 19.9] vs 19.8 [18.8, 20.7], p = 0.28
Visser (2023)	Belgium	Crossover follow-up to Visser 2021, n = 229, 104 weeks	rt-rtCGM: 42.8 ± 13.8 years, 18 [10–30] years, F: 46 (36%) is-rtCGM: 43.0 ± 14.5 years, 17 [8–28] years, F: 51 (40%)	rt-rtCGM	is-rtCGM	HFS-II W and HFS-II B		Within-group change rtCGM score change from 0 months (start of rt CGM) to 24 months: HFS-II W: −5.17 points (p < 0.0001) HFS-II B: −1.04 point (p = 0.015) isCGM score change from 6 months (start of rt CGM) to 24 months: HFS-II W: −2.67 points (p = 0.0008) HFS-II B: −0.56 points (p = 0.18)
**Observational studies:**								
Boscari (2022)	Italy	Prospective observational study, n = 38, 8 weeks	33.7 ± 12.6 years, 21.2 ± 12.0 years, F: 21 (55%)	isCGM (with alarms)	isCGM (without alarms)	HFS-II T, HFS-II W and HFS-II B	HFS-II T: 43.8 ± 25.9 HFS-II B: 20.8 ± 10.2 HFS-II W: 22.9 ± 16.6	HFS-II T: 28.6 ± 19.4, p = 0.004 HFS-II B: 14.9 ± 9.5, p = 0.01 HFS-II W: 13.7 ± 11.5, p = 0.003
Charleer (2020)	Belgium	Prospective observational cohort, n = 1913, 52 weeks	45.8 ± 15.3 years, 20.0 ± 13.7 years, F: 882 (46%)	isCGM	CBG	HFS-II W	18.1 [15.7; 20.6],	Score change between baseline and after 12 months (Least-squares mean [95% CI]) 6 months: 17.9 [15.6; 20.2], p = 0.34 12 months: 17.8 [15.5; 20.1], p = 0.12
Charleer (2023)	Belgium	Prospective follow up to Charleer 2020, n = 1902, 104 weeks	IAH group: 51.6 ± 14.6 years, 30.0 ± 14.3 years, F: 131 (43%) NAH group: 44.7 ± 15.2 years, 21.5 ± 13.1 years, F: 747 (47%)	isCGM	CBG	HFS-II W	IAH group:22.8 [21.4; 24.2], NAH group:17.2 [16.7; 17.8],	Score change between baseline and after 12 months (Least-squares mean [95% CI]) IAH group: 12 months: 22.2 [20.6; 23.8], p = 0.37 24 months: 20.6 [19.0; 22.1], p = 0.002 NAH group: 12 months: 16.9 [16.3; 17.5], p = 0.14 24 months: 17.2 [16.5; 17.8], p = 0.79
Charleer (2018)	Belgium	Prospective multicenter cohort, n = 515, >52 weeks	42.2 ± 12.5 years, 22.3 ± 11.6 years, F: 299 (59%)	rtCGM	CBG	HFS-II W	18.6 ± 10.5	15.1 ± 9.5, p < 0.0005
Charleer (2020)	Belgium	Prospective follow up to Charleer 2020, n = 441, 104 weeks	41.9 ± 12.5 years22.3 ± 11.6 years, F: 257 (58%)	rtCGM	CBG	HFS-II W	Least-squares mean [95%CI]: 18.3 [16.7, 19.9]	12 months: 14.7 [13.2; 16.3], p < 0.0001 24 months: 14.1 [12.5; 15.7], p < 0.0001
Munshi (2022)	United States	Cross-sectional, n = 165, ≥26 weeks	70 ± 10 years, 40 ± 17 years, F: 116 (52%)	rtCGM	CBG	HFS-II T, HFS-II W and HFS-II B	NA	rtCGM vs CBG: HFS-T: 31.9 ± 20.1vs 26.2 ± 14.4 p = NS HFS-W: 15.0 ± 8.6 vs 13.3 ± 6.9 p = NS HFS-B: 16.9 ± 13.6 vs 12.9 ± 13.3 p = NS
Nefs (2019)	The Netherlands	Prospective survey, n = 60, 26 weeks	38 ± 11 years, 23 ± 11 years, F: 48 (80%)	rtCGM	CBG	HFS-W	19.55 ± 11.39	15.86 ± 11.00, p = 0.02
Rouhard (2020)	Belgium	Prospective observational study, n = 248, 72 weeks	45 ± 16 years, 23 ± 13 years, F: 114 (46%)	isCGM	CBG	HFS-II W and HFS-II B (short form)	HFS-W: 7.2 ± 4.8 HFS-B: 5.7 ± 4.1	HFS-W: 6.6 ± 5.4, p = 0.15 HFS-B: 4.4 ± 3.6, p < 0.001

CI, confidence interval; IQR, interquartile range; SE, standard error; NS, non-significant; rtCGM, Real-Time continuous glucose monitor; isCGM, intermittently scanned continuous glucose monitor; CBG, capillary blood glucose; CSII, continuous subcutaneous insulin infusion; HFS, hypoglycaemia fear survey; HFS-T, HFS-total scale; HFS-W, HFS- Worry subscale; HFS-B, HFS-Behaviour subscale; Swe-HFS, Swedish version of the HFS; RCT, randomized controlled trial; FOH, fear of hypoglycaemia. IAH, impaired hypoglycaemia awareness; NAH, Normal hypoglycaemia awareness; NA: not applicable, NS: not significant; F, female particpants.

Hypoglycaemia fear survey scale and subscales: high scores reflect high levels of fear.

a Data presented as mean ± standard deviation or median [interquartile range] unless stated otherwise.

b 2 × 2 factorial RCT assessing both CSII and CGM at the same time.

**Table 2:** Studies assessing the impact of insulin administration modes on fear of hypoglycaemia as a measurable outcome among adults with type 1 diabetes.

Study	Location	Study design, sample size, duration of intervention	Baseline subject characteristics (age, diabetes duration, Female n (%))	Technology assessed	Comparison	Tools used to assess FOH	Baseline FOH score	Results^a^
**Experimental studies:**								
Heller (2017)	United Kingdom	Cluster RCT, n = 267, 104 weeks	Intervention arm: 41.5 ± 14.2 years, 18.5 ± 12.9 years, F: 54 (41%) Control arm: 39.9 ± 12.5 years, 17.5 ± 12.1 years, F: 53 (39%)	CSII	MDI	HFS-B and HFS-W	CSII vs MDI: HFS-W: 40.7 ± 14.6 vs 37.9 ± 13.3 HFS-B: 30.3 ± 5.8 vs 29.1 ± 5.5	Mean difference from baseline to 24 months CSII vs MDI: HFS-W: −6.7 ± 13.0 vs −2.9 ± 12.5, p = 0.01 HFS-B: −1.4 ± 5.6 vs −0.6 ± 5.1, p = 0.44
Little (2014)^b^	United Kingdom	2 × 2 RCT, n = 96, 24 weeks	49 ± 12 years, 29 ± 12 years, F: 66 (64%)	CSII	MDI	HFS-II T, HFS-II W and HFS-II B	HFS-T: 58 ± 26 HFS-W: 35 ± 17 HFS-B: 24 ± 11	CSII vs MDI: HFS-T: 44 ± 23 vs 45 ± 25, p = 0.82 HFS-W: 24 ± 17 vs 25 ± 17, p = 0.99 HFS-B: 20 ± 10 vs 21 ± 10, p = 0.61
Thomas (2007)	United Kingdom	RCT, n = 21, 24 weeks	43 ± 10 years, 25 ± 10 years, F: 10 (48%)	CSII	MDI	HFS T	CSII: 67 ± 19 Analog insulin: 91 ± 21	CSII: 64 ± 16, p = 0.21 Analog insulin: 83 ± 26, p = 0.06
**Observational studies:**								
Barnard & Skinner (2008)	United Kingdom	Cross-sectional, n = 642, 3.5 ± 2.8 years (182 weeks)	44.9 ± 13.5 years, 23.8 ± 12.4 years, F: 442 (69%)	CSII	MDI	HFS T, HFS W and HFS B	NA	CSII vs MDI: HFS-T: 33.9 ± 14.5 vs 40.5 ± 16.0, p < 0.001 HFS-B: 18.5 ± 5.1 vs 20.7 ± 6.0, p < 0.001 HFS-W: 15.1 ± 10.9 vs 20.0 ± 12.5, p < 0.001
Boulet (2016)	Canada	Cross-sectional, n = 305, >104 weeks	65 [59, 71] years, 54 [51, 59] years, F: 137 (55%)	CSII	MDI	HFS-II T, HFS-II W and HFS-II B	NA	CSII vs MDI: HFS-T: 26 [15, 41] vs 31 [17, 44], p = 0.14 HFS-B: 13 [8, 19] vs 15 [9, 21], p = 0.20 HFS-W: 11 [6, 23] vs 14 [7, 25], p = 0.24
Halbron (2019)	France	non-randomized trial, n = 17, 26 weeks	25 [20, 32] years, 13 [9, 17] years, F: 11 (58%)	CSII	MDI	HFS-T, HFS-W and HFS-B	HFS-T: 67 [51, 79] HFS-B: 25 [23, 31] HFS-W: 38 [29, 47]	HFS-T: 68 [57, 80], p = 0.46 HFS-B: 28 [26, 29], p = 0.13 HFS-W: 37 [28, 52], p = 0.86
Hohendorff (2022)	Poland	Cross-sectional, N = 77, No information on the duration of use	34.1 ± 10.2 years, 14.7 ± 12.0 years, F: 58 (56%)	CSII	MDI	HFS-II T, HFS-II W and HFS-II B	NA	CSII vs MDI: HFS II-T: 36.3 ± 16.7 vs 33.2 ± 16.9, p = 0.42 HFS II-B: 15.5 ± 6.2 vs 16.6 ± 8.0, p = 0.49 HFS II-W: 20.8 ± 13.4 vs 16.6 ± 10.7, p = 0.13
Linkeschova (2002)	Germany	Prospective observational study, n = 103, 104 weeks	33 ± 11 years, 17.6 ± 9 years, F: 58 (56%)	CSII	MDI	DSQOLS^c^	NA	CSII vs MDI: FOH score: 68% vs 54%, p < 0.001
McAuley (2021)	Australia	Cross-sectional, n = 120, 3 weeks	CSII arm 43 [36, 53] years, 24 [18, 31] years, F: 34 (58%) MDI arm 43 [35, 53] years, 24 [16, 31] years, F: 29 (48%)	CSII	MDI	HFS II (short form)	NA	CSII vs MDI: HFS-W: 7 (4, 12) vs 6 (4, 10), p = 0.40 HFS-B: 4 (2, 6) vs 4 (2, 6), p = 0.97
Munshi (2022)	United States	Cross-sectional, n = 165, ≥26 weeks	70 ± 10 years 40 ± 17 years F: 86 (52%)	CSII	MDI	HFS-II T, HFS-II W and HFS-II B	NA	CSII vs MDI: HFS-T: 26.8 ± 16.7 vs 33.4 ± 19.1, p = 0.03 HFS-W: 13.6 ± 8.1 vs 15.3 ± 7.3, p = NS HFS-B: 13.1 ± 11.1 vs 18.1 ± 14.1, p = 0.03
Nicolucci (2008)	Italy	Non-randomized case-control study, n = 1341, ≥26 weeks	CSII arm 35.1 ± 10.9 years, 18.4 ± 10.2 years, F: 275 (57.%) MDI arm 34.9 ± 12.4 years, 14.9 ± 9.8 years, F: 393 (46%)	CSII	MDI	DSQOLS^c^	NA	CSII vs MDI: Age, gender, and diabetes duration-adjusted mean scores: 66.3 ± 1.10 vs 63.9 ± 0.81, p = 0.08 Raw scores: 65.9 ± 23.0 vs 63.9 ± 24.4, p = 0.25
Perez-Garcia (2015)	Spain	Retrospective two-arm study, n = 60, 52 weeks	CSII arm No age reported: 16.30 [12.91–19.69] years, MDI ram No age reported: 18.13 [15.38–20.88] years In both groups, F: 38 (63%)	CSII	MDI	FH-15	NA	CSII vs MDI: 26.88 ± 7.17 vs 28.04 ± 7.87, p = 0.49
Scheidegger (2007)	Switzerland	Cross-sectional, n = 159, 2.1 ± 2.6 years (104 weeks)	CSII arm: 41.3 ± 13.3 years, 19.4 ± 11.6 years, F: 37 (47%) MDI arm: 42.2 ± 11.8 years, 17.4 ± 10.6 years, F: 39 (48%)	CSII	MDI	DSQOLS^c^	NA	CSII vs MDI: Worries about hypoglycaemia score^d^: 47 ± 12 vs 46 ± 14, p = NS
		Prospective longitudinal study, n = 19, 17–26 weeks	42.8 ± 11.2 years, 18.2 ± 11.0 years, F: 19 (74%)	CSII	MDI	DSQOLS^c^	NA	CSII vs MDI: Worries about hypoglycaemia score: 41 ± 17 vs 48 ± 13, p = NS
Shaban (2017)	United Kingdom	Prospective cohort, n = 45, 52 weeks	39.6 ± 13.9 years, 21.0 ± 14.1 years, F: 42%	CSII	MDI	HFS-II W and HFS-II B	HFS-W: 24.1 ± 16.3 HFS-B: 19.1 ± 9.7	HFS-W: 15.0 ± 12.9, p < 0.001 HFS-B: 13.4 ± 8.1, p < 0.001

CI, confidence interval; IQR, interquartile range; SE, standard error. CSII, continuous subcutaneous insulin infusion; MDI, multiple daily injections; HFS, hypoglycaemia fear survey; HFS-W, HFS- Worry subscale; HFS-B, HFS-Behaviour subscale; HFS-T, HFS-Total scale; DSQOLS, Diabetes-Specific Quality-of-Life Scale; FH-15, fear of hypoglycaemia 15-item scale; RCT, randomized controlled trial; FOH, fear of hypoglycaemia. NA: not applicable, NS: not significant; F, female particpants.

a Data presented as mean ± standard deviation or median [interquartile range] unless stated otherwise.

b 2 × 2 factorial RCT assessing both CSII and CGM at the same time.

c A higher value means the DSQOLS parameter is less of a burden.

d Adjusted for age, gender, duration of diabetes, HbA1c, presence or absence of retinopathy, professional situation (working, disabled, retired), accommodation (alone, with partner, with children, other). Hypoglycemia fear survey scale and subscales: high scores reflect high levels of fear.

**Table 3:** Studies assessing the impact of combining integrated insulin administration and continuous glucose monitoring technologies on fear of hypoglycaemia as a measurable outcome among adults with type 1 diabetes.

Study	Location	Study design, sample size, duration of technology use	Baseline subject characteristics (age, diabetes duration, Female n (%))	Technology assessed	Comparison	Tools used to assess FOH	Baseline FOH score	Results^a^
**Experimental trials:**								
Bisio (2021)	United States	Non-randomized single arm with two treatment periods, n = 15, 4 weeks each period	68.7 ± 3.3 years, 35.2 ± 12.6 years, F: 6 (40%)	AID	SAP	HFS-II T, HFS-II W and HFS-II B	HFS-T: 26.29 ± 14.60 HFS-W: 12.5 ± 9.95 HFS-B: 13.79 ± 5.63	AID vs SAP phase: HFS-T: 27.64 ± 14.11 vs 24.21 ± 12.45, p = NS HFS-W: 13.07 ± 9.43 vs 10.71 ± 8.59, p = NS HFS-B: 14.57 ± 5.69 vs 13.5 ± 5.43, p = NS
Burckhardt (2021)	Australia	Randomized crossover (pilot), n = 15, 8 weeks each period	35.8 ± 11.2 years, 24.2 ± 11.3 years, F: 12 (71%)	AID	CSII	HFS-II T and HFS-II W	NA	After AID vs CSII phase: HFS-T: 46.5 [36.5, 71.0] vs 61.5 [47.5, 77.0], p = 0.43 HFS-W: 35.5 [29.5, 49.5] vs 46.5 [34.5, 55.5], p = 0.22
Choudhary (2022)	Europe	RCT, n = 74, 26 weeks	AID: 41.5 ± 11.6 years, 18.8 ± 11.4 years, F: 22 (54%) MDI + isCGM: 39.7 ± 13.1 years, 18.1 ± 9.97 years, F: 16 (39%)	AID	MDI + isCGM	HFS-T, HFS-B and HFS-W	AID vs MDI + isCGM: HFS-T: 44.0 ± 19.13 vs 50.9 ± 21.16 HFS-B: 20.4 ± 7.22 vs 23.0 ± 8.95 HFS-W: 23.7 ± 14.27 vs 27.9 ± 14.63	AID vs MDI + isCGM: HFS-T: 35.7 ± 23.38 vs 47.4 ± 22.88, p = 0.041 HFS-B: 15.8 ± 9.47 vs 21.8 ± 8.59, p = 0.047 HFS-W: 19.9 ± 15.26 vs 25.6 ± 15.98, p = 0.18
Kropff (2017)	Europe	Crossover RCT, n = 31, 8 weeks	47 ± 11.2 years, 28.6 ± 10.8 years, F: 18 (56%)	SAP + overnight AID	SAP all day	HFS-II T, HFS-II W and HFS-II B	HFS-T: 28.2 ± 17.5 HFS-W: 14.9 ± 11.8 HFS-B: 14.0 ± 7.6	SAP + overnight AID phase: HFS-T: 23.5 ± 16.7 HFS-W: 11.7 ± 10.1 HFS-B: 12.2 ± 8.1 SAP all day phase: HFS-T: 23.5 ± 16.6, p = 0.099 HFS-W: 11.6 ± 9.9, p = 0.074 HFS-B: 12.4 ± 7.8, p = 0.27
Kudva (2021)	United States	RCT, n = 120, 26 weeks	AID: 33 ± 16 years, 17 [8, 28] years, F: 54 (48%) SAP: 33 ± 17 years, 15 [7, 23] years, F: 30 (54%)	AID	SAP	HFS-II T, HFS-II W and HFS-II B	AID vs SAP HFS T: 39 ± 14 vs 42 ± 18 HFS B: 51 ± 12 vs 55 ± 16 HFS W: 29 ± 18 vs 32 ± 23	AID vs SAP HFS T: 33 ± 12 vs 38 ± 18, p = 0.23 HFS B: 43 ± 12 vs 52 ± 15, p = 0.02 HFS W: 25 ± 15 vs 27 ± 22, p = 0.80
McAuley (2022)	Australia	Crossover RCT, n = 30, 17 weeks each period	67 ± 5 years, 38 [20, 47] years, F: 19 (63%)	AID	SAP	HFS II (short form)	HFS-W: 5 [3,10] HFS-B: 3.5 [2, 5]	After AID vs SAP phase: HFS-T: 7.5 (4, 10) vs 7.5 (5, 10), p = 0.72 HFS-W: 4.5 (2, 7) vs 4.5 (3, 7), p = 0.14 HFS-B: 2 (1, 4) vs 2 (1, 4), p = 0.087
Wheeler (2022)	New Zealand	Crossover RCT, n = 28, 4 weeks each period	18–65 years, No T1D duration, F: 35 (58%)	AID	SAP	HFS-II W and HFS-II B	NA	After AID vs SAP phase (mean (SE)): HFS-B: 1.13 (0.08) vs 1.26 (0.08), p = 0.19 HFS-W: 1.01 (0.11) vs 1.10 (0.11), p = 0.57
Hermanides (2011)	Europe	RCT, n = 83, 26 weeks	SAP: 39.3 ± 11.9 years, 16.9 ± 10.7 years, F: 22 (50%) MDI: 37.3 ± 10.7 years, 21.0 ± 9.4 years, F: 18 (46%)	SAP	MDI	HFS-W	SAP vs MDI: 29.8 ± 19.2 vs 21.0 ± 17.7	SAP vs MDI: 24.1 ± 20.2 vs 20.3 ± 16.9, p = 0.42
Schmidt (2012)	Europe	Follow up of Hermanides (2011), n = 24, 156 weeks	39.0 ± 11.9 years, 20.3 ± 10.3 years, F: 12 (50%)	SAP	MDI	HFS-T	25.8 ± 19.2	20.3 ± 14.7, p = 0.15
Rubin (2012)	United States	RCT, n = 334, 52 weeks	41.3 ± 12.3 years 20.2 ± 12.0 years, F: 142 (43%)	SAP	MDI	HFS-II W and HFS-II B	SAP vs MDI: HFS-B: 16.38 ± 8.24 vs 16.70 ± 8.00 HFS-W: 21.96 ± 4.34 vs 21.52 ± 13.37	Change in scores, from baseline to end of study: Control: HFS-W: −1.87, p > 0.05 HFS-B: −0.52, p > 0.05 Intervention HFS-W: −6.36, p < 0.001 HFS-B: −2.30, p < 0.001
Bosi (2019)	Canada, France, Italy, the Netherlands, and the UK	Open-label RCT, n = 153, 26 weeks	SAP: 49.0 ± 12.2 years, 28.5 ± 11.1 years, F: 38 (50%) CSII: 47.4 ± 12.5 years, 29.7 ± 13.3 years, F: 38 (49%)	SAP	CSII	HFS-T, HFS-B, HFS-W	SAP vs CSII: HFS-T: 52.0 ± 23.2 vs 46.7 ± 20.7 HFS-B: 20.0 ± 9.9 vs 19.0 ± 9.2 HFS-W: 32.0 ± 15.9 vs 27.6 ± 13.8	SAP vs CSII: Raw scores: HFS-T: 33.7 ± 19.0 vs 38.9 ± 23.4 HFS-B: 15.5 ± 8.7 vs 17.5 ± 10.0 HFS-W: 18.2 ± 13.2 vs 21.4 ± 15.3 Score change from baseline: HFS-T: −17.6 ± 22.1 vs −7.2 ± 14.9; p = 0.001 HFS-B: −4.1 ± 9.8 vs −1.3 ± 6.7; p = 0.04 HFS-W: −13.5 ± 15.0 vs −5.9 ± 10.3; p = 0.0004
**Observational studies:**								
Boscari (2022)	Italy	Retrospective observational study, n = 31, 12 weeks	38 [31, 45] years, 16 [15–26] years, F: 14 (45%)	AID (control IQ)	SAP	HFS-II T, HFS-II W and HFS-II B	HFS-T: 37.4 ± 22.0 HFS B: 17.5 ± 9.3 HFS W: 19.8 ± 14.3	HFS-T: 26.3 ± 16.1, p = 0.01 HFS B: 13.0 ± 8.0, p = 0.02 HFS W: 13.2 ± 9.3, p = 0.01
Murata (2021)	Japan	Prospective observational study, n = 43, 52 weeks	44.1 ± 15.0 years, 18.8 ± 13.6 years, F: 34 (74%)	SAP	CSII	HFS-II T, HFS-II B, HFS-II W	HFS-T: 34.3 ± 16.8 HFS-B: 16.9 ± 6.6 HFS-W: 17.3 ± 12.0	HFS-T: 30.3 ± 13.7, p = 0.06 HFS-B: 16.9 ± 6.8, p = 0.95 HFS-W: 13.5 ± 8.9, p = 0.02
Norgaard (2013)	Europe	Observational cohort, n = 179, 52 weeks	39.2 ± 10.2 years, 19.3 ± 10.5 years, F: 119 (67%)	SAP	CSII	HFS-II T, HFS-II B, HFS-II W	HFS-T: 37.0 ± 26.0 HFS-B: 16.03 ± 10.6 HFS-W: 23.39 ± 15.7	HFS-T: 28.95 ± 20.1, p = 0.003 HFS-B: 13.35 ± 8.9, p = 0.02 HFS-W: 18.4 ± 14.9, p = 0.003
Wu (2020)	China	Retrospective cohort, n = 15, 13 weeks	32.0 [19.2, 69.4] years, 9.7 [1.8, 23.7] years, F: 10 (67%)	AID	SAP	HFS-II W	26.27 ± 5.82	22.13 ± 6.87, p = 0.01

CI, confidence interval; IQR, interquartile range; SE, standard error. CGM, continuous glucose monitor; CBG, capillary blood glucose; CSII, continuous subcutaneous insulin infusion; SAP, Sensor-augmented pump; HFS, hypoglycaemia fear survey; HFS-W, HFS-Worry subscale; HFS-B, HFS-Behaviour subscale; RCT, randomized controlled trial; FOH, fear of hypoglycaemia. NA: not applicable; F, female particpants.

Hypoglycaemia fear survey scale and subscales: high scores reflect high levels of fear.

a Data presented as mean ± standard deviation or median [interquartile range] unless stated otherwise.

The statistical analyses were conducted using the meta package (version 6.2-0) and metafor (version 3.8-1) in R (version 4.1.2). Standardized mean differences (Hedges g) (SMD) with 95% confidence intervals (CIs) were calculated to evaluate the change in FOH. As the studies used different measurement tools (such as HFS or HFS-II) to quantify the level of FOH, we elected to use the SMD as a summary statistic to standardize the results to a uniform scale so we can effectively combine the findings in this analysis. When the mean and standard deviation were not available, we either contacted the authors, converted the available statistic to mean and standard deviation (when appropriate), or did not include the study in the synthesis. Our primary outcome was the difference or change in FOH (total FOH measured with validated surveys) and secondary outcomes were change in HFS-B or HFS-W scores when applicable. Outcomes were compared between the intervention (technology use such as rtCGM, CSII, or AID use) and the control conditions (such as MDI or CBG). Specifically, the following comparisons were explored: rtCGM (intervention) vs isCGM or CBG (comparator or control), isCGM vs CBG, CSII vs MDI, SAP vs MDI or CSII, and AID vs MDI, or CSII or SAP.

Studies included in the meta-analysis were divided into two groups, 1) RCTs were synthesized together in one analysis and 2) non-RCTs; remaining studies with either a prospective or retrospective design were synthesized in a separate analysis (cross-sectional studies were not included in the meta-analysis).^25^ The Cochrane chi-squared test and I^2^ index were used to evaluate heterogeneity between the articles. Random effects models were used as determined pre-analysis even when I^2^<50%. Subgroup analyses were performed according to the technology used, the duration of its use (<26 weeks vs ≥26 weeks), and if the frequency of hypoglycaemia was significantly decreased or not. A leave-one-out sensitivity analysis was also performed to evaluate the robustness of the meta-analysis (for RCT and non-RCT analysis, separately). Publication bias was assessed by visual inspection of the symmetry in funnel plots and using Egger’s test, which was conducted separately for RCT and non-RCT studies via Statistical Package for Social Sciences (SPSS) V25.

### Role of the funding source

The funders of the study had no role in study design, data collection, data analysis, data interpretation, or writing of the report. M.K.T., A.K., L.H., and A.S.B had access to the dataset and had final responsibility for the decision to submit for publication.

## Results

**Fig. 1:**
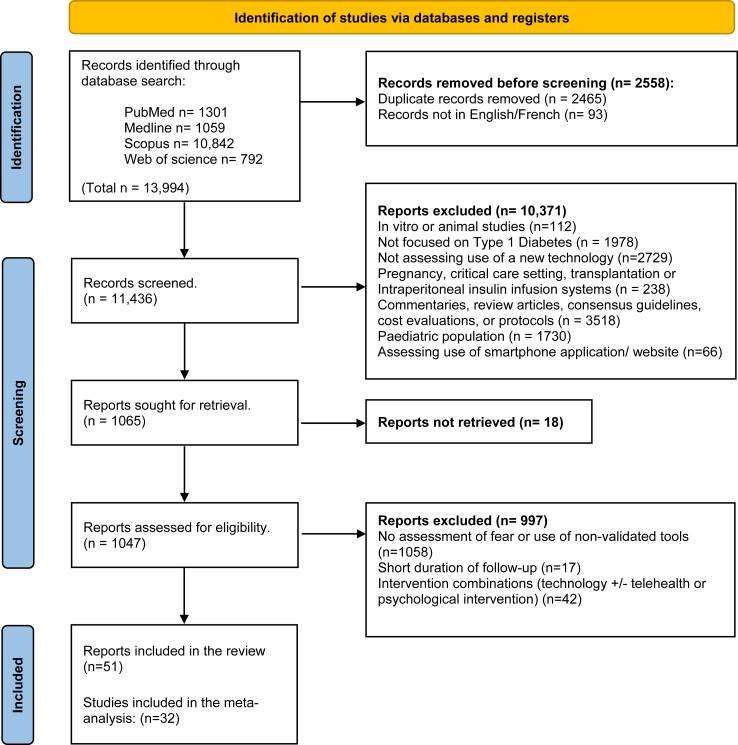
Flow diagram of the article selection process (PRISMA 2020).

More than 13,000 articles were retrieved and exported to EndNote. Duplicates were eliminated, followed by an initial screening of article titles and abstracts. The remaining 1047 articles were reviewed in full, and inclusion and exclusion criteria were applied, after which 51 articles involving 8966 adult participants remained for inclusion in this review (Fig. 1).

Study sample sizes ranged between 15 and 1913 participants. The male-to-female distribution ranged between 32% and 80% female. The follow-up period ranged between 4 and 156 weeks (i.e., 1–36 months). Studies with the highest participant numbers and longest follow-up periods were of an observational nature.^26^, ^27^, ^28^ Of the included articles, 22 were RCTs,^29^, ^30^, ^31^, ^32^, ^33^, ^34^, ^35^, ^36^, ^37^, ^38^, ^39^, ^40^, ^41^, ^42^, ^43^, ^44^, ^45^, ^46^, ^47^, ^48^, ^49^, ^50^ two non-randomized trials,^51^^,^^52^ 13 longitudinal observational studies,^27^^,^^28^^,^^53^, ^54^, ^55^, ^56^, ^57^, ^58^, ^59^, ^60^, ^61^, ^62^, ^63^ six cross-sectional studies,^26^^,^^64^, ^65^, ^66^, ^67^, ^68^ and eight follow-up reports.^69^, ^70^, ^71^, ^72^, ^73^, ^74^, ^75^, ^76^ Most of the studies used the HFS or HFS-II to measure FOH (91%). The majority of the studies were conducted in Europe (n = 22, 51%),^27^^,^^28^^,^^30^^,^^33^^,^^34^^,^^36^^,^^37^^,^^39^^,^^42^^,^^48^^,^^49^^,^^52^, ^53^, ^54^, ^55^, ^56^^,^^58^, ^59^, ^60^, ^61^^,^^65^^,^^68^ the United States (n = 7, 16%),^29^^,^^38^^,^^43^^,^^44^^,^^46^^,^^51^^,^^67^ and the United Kingdom (n = 6, 14%).^26^^,^^35^^,^^40^^,^^45^^,^^47^^,^^62^ Study characteristics, descriptions of the interventions and comparison groups, and main findings are summarized in Table 1, Table 2, Table 3.

Table 1 describes the studies assessing the impact of glucose monitoring technologies on FOH as a measurable outcome among adults with T1D. Out of the 24 reports, 18 reported on rtCGM use^29^^,^^34^^,^^39^^,^^40^^,^^43^, ^44^, ^45^^,^^48^^,^^49^^,^^55^^,^^58^^,^^67^^,^^70^, ^71^, ^72^, ^73^, ^74^^,^^76^ and six on isCGM.^27^^,^^30^^,^^42^^,^^54^^,^^61^^,^^69^ Of the 18 rt-CGM reports, 12 were primary studies^29^^,^^34^^,^^39^^,^^40^^,^^43^, ^44^, ^45^^,^^48^^,^^49^^,^^55^^,^^58^^,^^67^ (9 RCTs) and 6 were follow-ups.^70^, ^71^, ^72^, ^73^, ^74^^,^^76^ The use of rtCGM was found to significantly reduce FOH in four RCTs after at least 16 weeks of use,^29^^,^^45^^,^^48^^,^^49^ in two prospective observational studies after 26 weeks of use,^34^^,^^58^ and in three follow-ups ranging between 52 and 104 weeks.^70^^,^^72^^,^^76^ Among the six reports on isCGM use, five were primary studies^27^^,^^30^^,^^42^^,^^54^^,^^61^ (two RCTs and three prospective cohorts) and one was a follow-up report.^69^ None of the RCTs showed a significant reduction in FOH after isCGM use for 26 weeks.^30^^,^^42^ However, among the observational studies, two showed a significant decrease in FOH scores.^54^^,^^61^ It is worth noting that one of these studies compared participants using isCGM with alarms as an intervention to those using isCGM without alarms as a control,^54^ while the other study reported a significant decrease only in the behaviour subscale of FOH.^61^ Furthermore, the follow-up study conducted by Charleer et al. revealed a decrease in FOH, but only after 24 months of follow-up and specifically among participants with impaired awareness of hypoglycaemia (IAH).^69^

Fourteen studies assessed the impact of using CSII on FOH (Table 2),^26^^,^^28^^,^^35^^,^^40^^,^^47^^,^^52^^,^^56^^,^^60^^,^^62^^,^^64^, ^65^, ^66^, ^67^, ^68^ of which three were RCTs.^35^^,^^40^^,^^47^ One RCT found a significant decrease in HFS-W scores after 104 weeks of CSII use compared to MDI.^35^ Of the observational studies (n = 11), four studies reported a statistically significant association between CSII use for ≥26 weeks and decreased FOH compared to MDI.^26^^,^^56^^,^^62^^,^^67^ In a cross-sectional study, PWT1D using CSII for an average of 3.5 ± 2.8 years reported lower FOH overall, less hypoglycaemia-related worry and lower adoption of compensatory behaviours compared to MDI.^26^ However, after controlling for socioeconomic status and frequency of BG monitoring, only results for the HFS-T and HFS-B scores remained significant.^26^

Table 3 describes the studies assessing the impact of AID or SAP use on FOH. Of the 15 reports, 14 were primary studies^31^, ^32^, ^33^^,^^36^, ^37^, ^38^^,^^41^^,^^46^^,^^50^^,^^51^^,^^54^^,^^57^^,^^63^ and one was a follow-up.^75^ Two out of the three RCTs comparing FOH change after SAP use to MDI or CSII with CBG found a significant decrease after at least 26 weeks of use.^31^^,^^46^ Similar results were also reported in observational studies by both Norgaard et al., and Murata et al., who found a significant decrease in HFS scores after 52 weeks of use compared to CSII.^57^^,^^59^ The two other reports evaluating SAP use showed no improvement in FOH even after a follow-up lasting 156 weeks.^36^^,^^75^ The remaining nine studies evaluated the impact of using AID technology on FOH.^32^^,^^33^^,^^37^^,^^38^^,^^41^^,^^50^^,^^51^^,^^54^^,^^63^ Of the six RCTs,^32^^,^^33^^,^^37^^,^^38^^,^^41^^,^^50^ only two showed evidence of lower FOH after 26 weeks of AID use compared to either MDI or SAP as controls.^33^^,^^38^ The two observational studies showed a significant decrease in FOH after 13 weeks of AID use compared to SAP.^53^^,^^63^ The remaining trials found no significant reduction in FOH compared to SAP^37^^,^^41^^,^^50^^,^^51^ or CSII.^32^

**Fig. 2:**
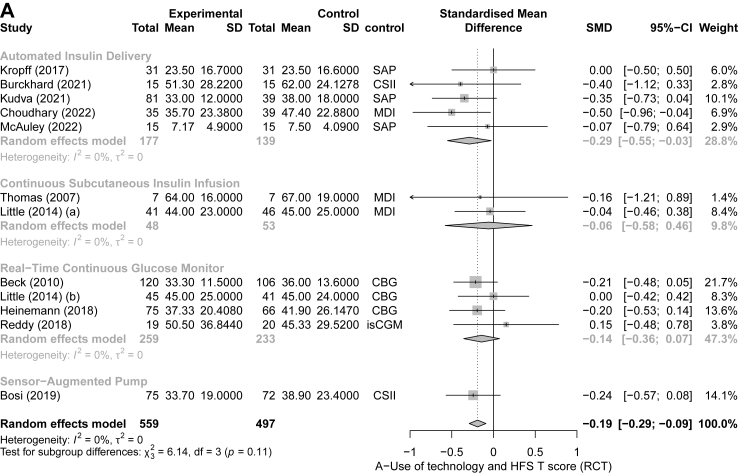

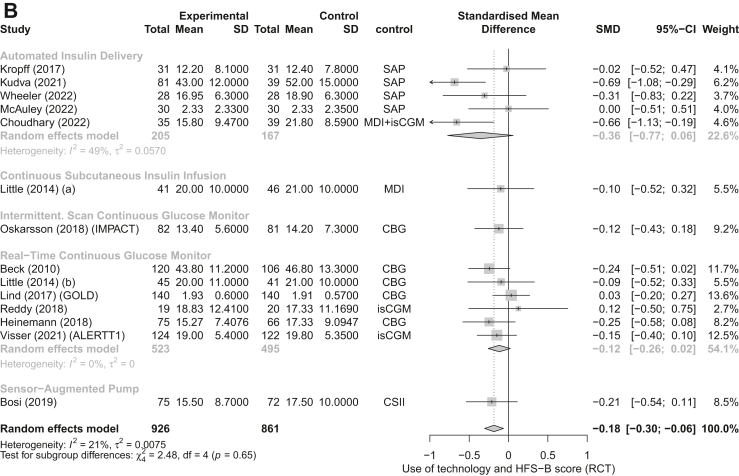

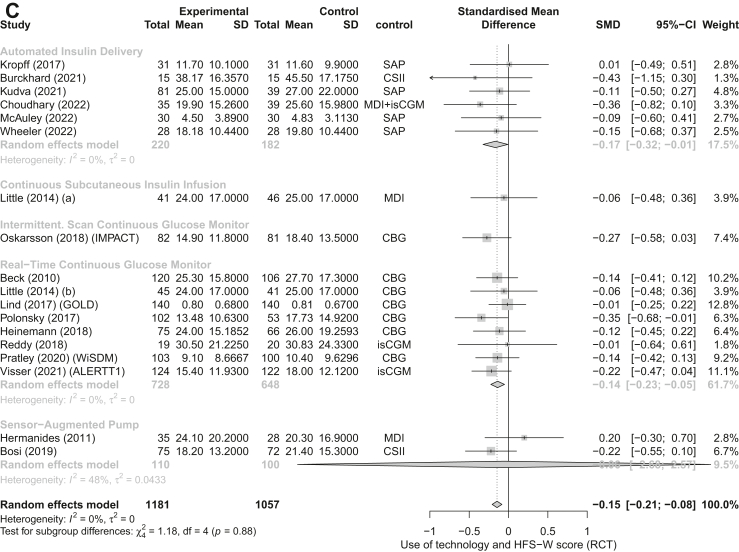
Forest plot representing the pooled estimated SMD of change in FOH between intervention and control methods in terms of HFS-T scale (A), HFS-B scale (B), HFS-W scale (C): randomized controlled trials (RCT). Footnotes: SD, standard deviation; CI, confidence interval; SMD, standard mean difference; rtCGM, real-time continuous glucose monitor; isCGM, intermittently scanned continuous glucose monitor; CBG, capillary blood glucose; MDI, multiple daily injections; CSII, Continuous Subcutaneous Insulin Infusion; SAP, sensor-augmented pump; HFS, hypoglycaemia fear survey; HFS-T, HFS-total scale; HFS-W, HFS-Worry subscale; HFS-B, HFS-Behaviour subscale; RCT, randomized controlled trial.

**Fig. 3:**
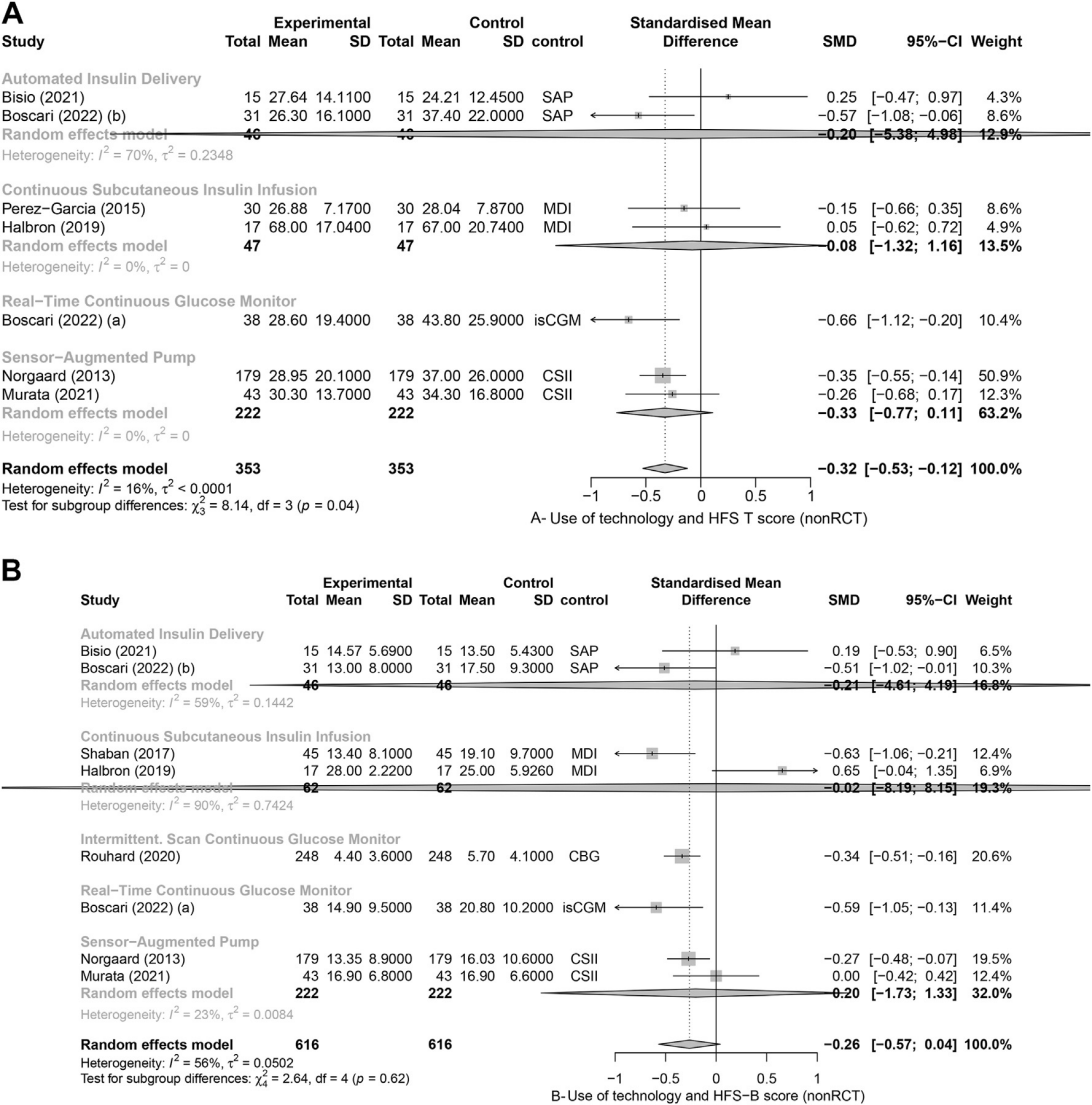

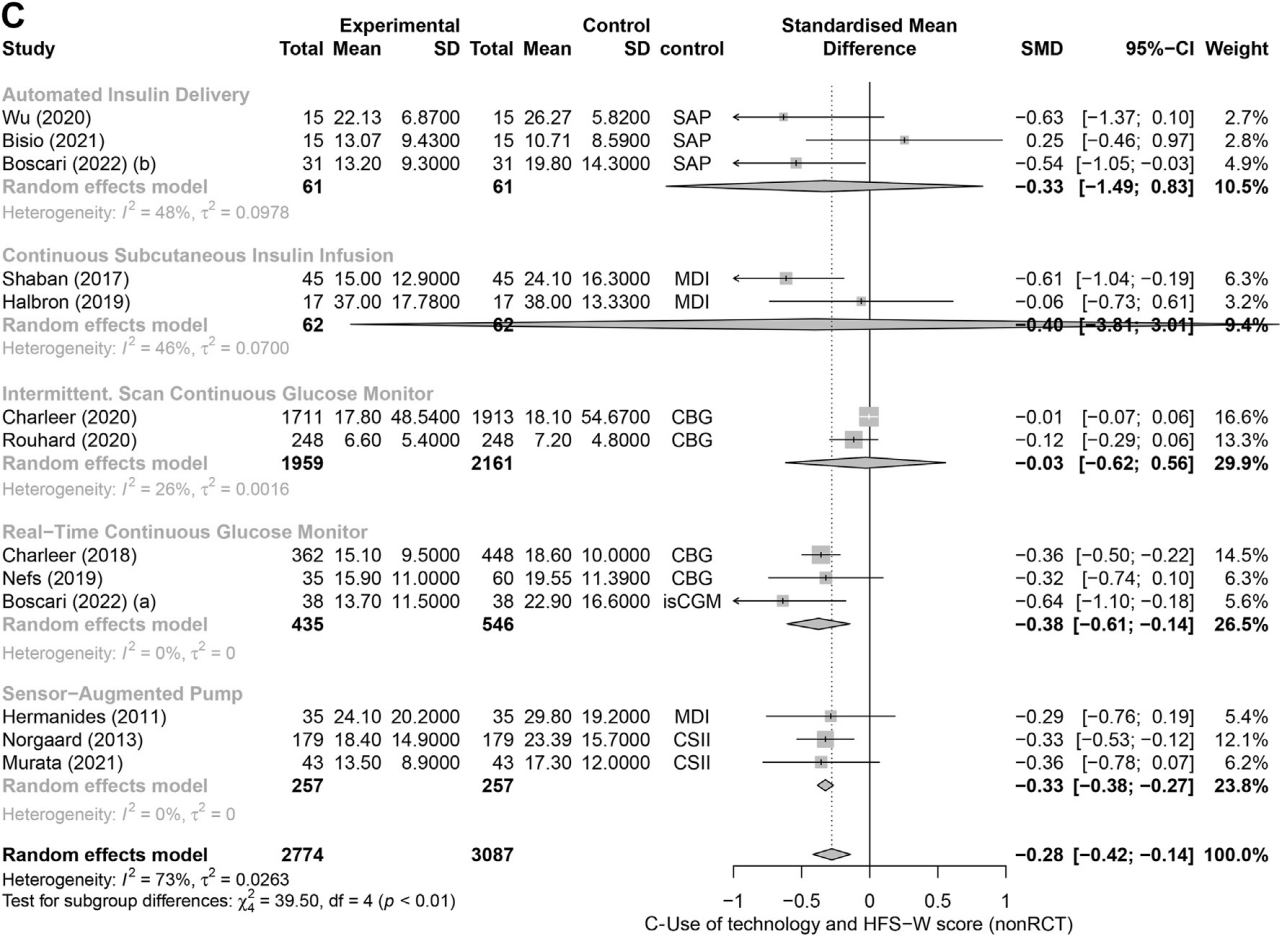
Forest plot representing the pooled estimated SMD of change in FOH between intervention and control methods in terms of HFS-T scale (A), HFS-B scale (B), HFS-W scale (C): non-randomized controlled trials (non-RCT). Footnotes: SD, standard deviation; CI, confidence interval; SMD, standard mean difference; rtCGM, real-time continuous glucose monitor; isCGM, intermittently scanned continuous glucose monitor; CBG, capillary blood glucose; MDI, multiple daily injections; CSII, Continuous Subcutaneous Insulin Infusion; SAP, sensor-augmented pump; HFS, hypoglycaemia fear survey; HFS-T, HFS-total scale; HFS-W, HFS-Worry subscale; HFS-B, HFS-Behaviour subscale; RCT, randomized controlled trial.

We included 18 RCTs in the RCT meta-analyses^29^^,^^31^, ^32^, ^33^, ^34^^,^^36^, ^37^, ^38^, ^39^, ^40^, ^41^, ^42^, ^43^, ^44^, ^45^^,^^47^^,^^49^^,^^50^ and 14 non-RCTs in the non-RCT meta-analysis.^27^^,^^36^^,^^51^, ^52^, ^53^, ^54^, ^55^^,^^57^, ^58^, ^59^, ^60^, ^61^, ^62^, ^63^ Eleven RCTs reported on the change in HFS-T scores. The forest plot analysis for RCTs showed that overall, the HFS-T scores were lower among technology users compared to controls (SMD = −0.19; 95% CI [−0.30, −0.08], I^2^ = 0%), specifically, when using AID technology (−0.29 [−0.55, −0.03]) (Fig. 2A). Similarly, the forest plot analysis for the seven non-RCT studies showed overall lower HFS-T scores amongst technology users (−0.32 [−0.53, −0.12]; I^2^ = 16%) (Fig. 3A). Thirteen RCTs and eight non-RCTs reported a change in HFS-B score. The forest plot analysis for RCTs showed that diabetes technology users had significantly lower HFS-B scores compared to control groups (−0.18 [−0.31, −0.05]; I^2^ = 32%) (Fig. 2B). The forest plot analysis of non-RCT studies showed that while there was a tendency for lower scores amongst technology users, the results were not statistically significant (−0.26 [−0.57, 0.04]; I^2^ = 56%) (Fig. 3B). Seventeen RCT and thirteen non-RCT studies reported the change in HFS-W scores. The forest plot analyses of RCT (Fig. 2C) and the forest plot analyses for nonRCTs (Fig. 3C) both showed that compared to control groups, users of diabetes technology had significantly lower HFS-W scores (−0.15 [−0.21, −0.09]; pI^2^ = 0% and (−0.28 [−0.42, −0.14]; I^2^ = 73%), respectively). In the subgroup analysis by type of technology, rt-CGM use was significantly associated with lower HFS-W subscale scores in both analysis of RCT (−0.14; [−0.23, −0.05], Fig. 2C); and analysis of non-RCT (−0.35; [−0.49, −0.22], Fig. 3C); studies. Meanwhile, AID use (−0.17; [−0.33, −0.01]) showed a significant decrease in the RCT analysis (Fig. 3B) and SAP use (−0.33; [−0.38, −0.27]) showed a significant decrease in the non-RCT analysis (Fig. 3C). Although mostly significant, the effect sizes ranged from small (mainly in the RCT meta-analysis) to moderate.

The test for subgroup differences between studies reporting less hypoglycaemia (a decrease in frequency or decreased time below range) and those that found no significant difference or did not report on hypoglycaemia found no statistically significant subgroup effect (p ≥ 0.14), suggesting that reduction in hypoglycaemia does not modify the effect of intervention in comparison to controls (Supplemental Figs. S1 and S2). Additionally, the subgroup difference analyses found no difference between the studies depending on the duration of technology use (<26 vs ≥26 weeks) (Supplemental Figs. S3 and S4). Except for the HFS-T scores’ subgroup analysis, which shows a statistically significant subgroup effect (p < 0.01) in the RCT studies, suggesting that a longer duration of technology use might be an effect modifier associated with the reduction in HFS-T scores compared to studies in which duration of technology use was <26 weeks (Supplemental Figs. S3A).

**Fig. 4:**
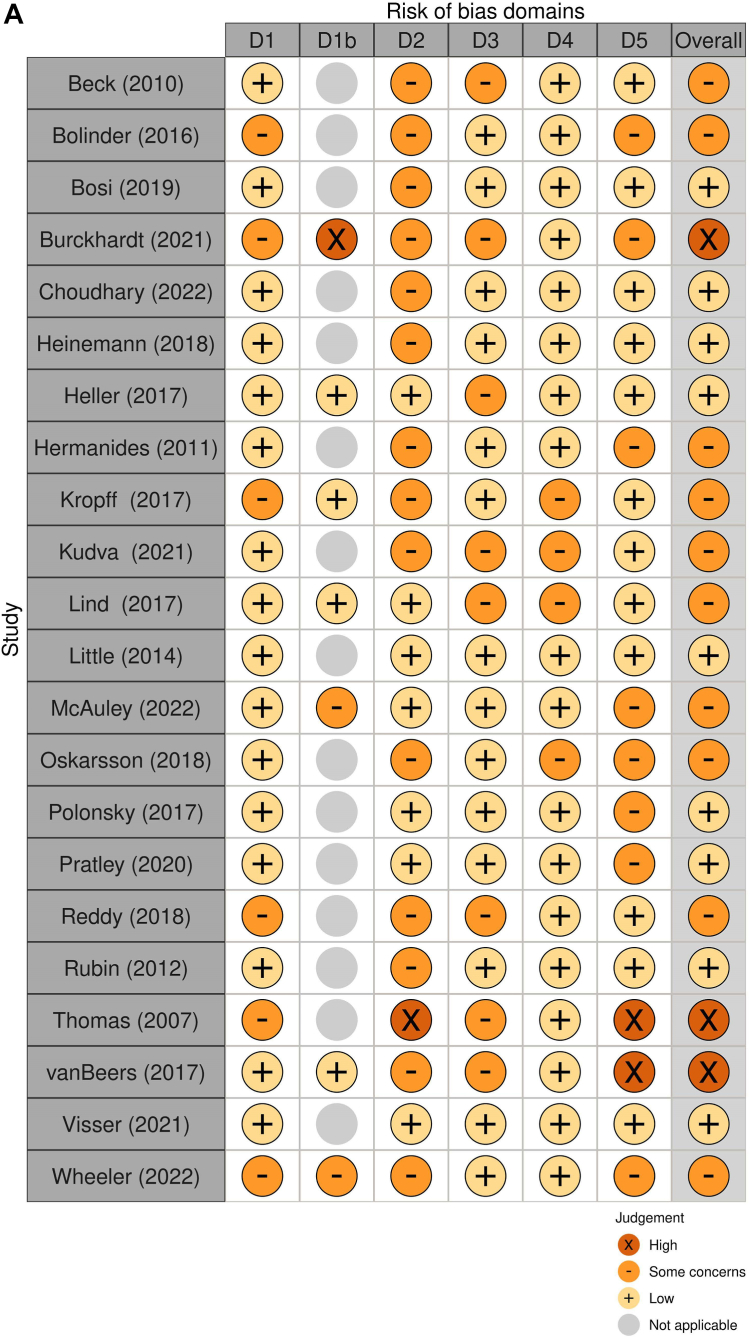

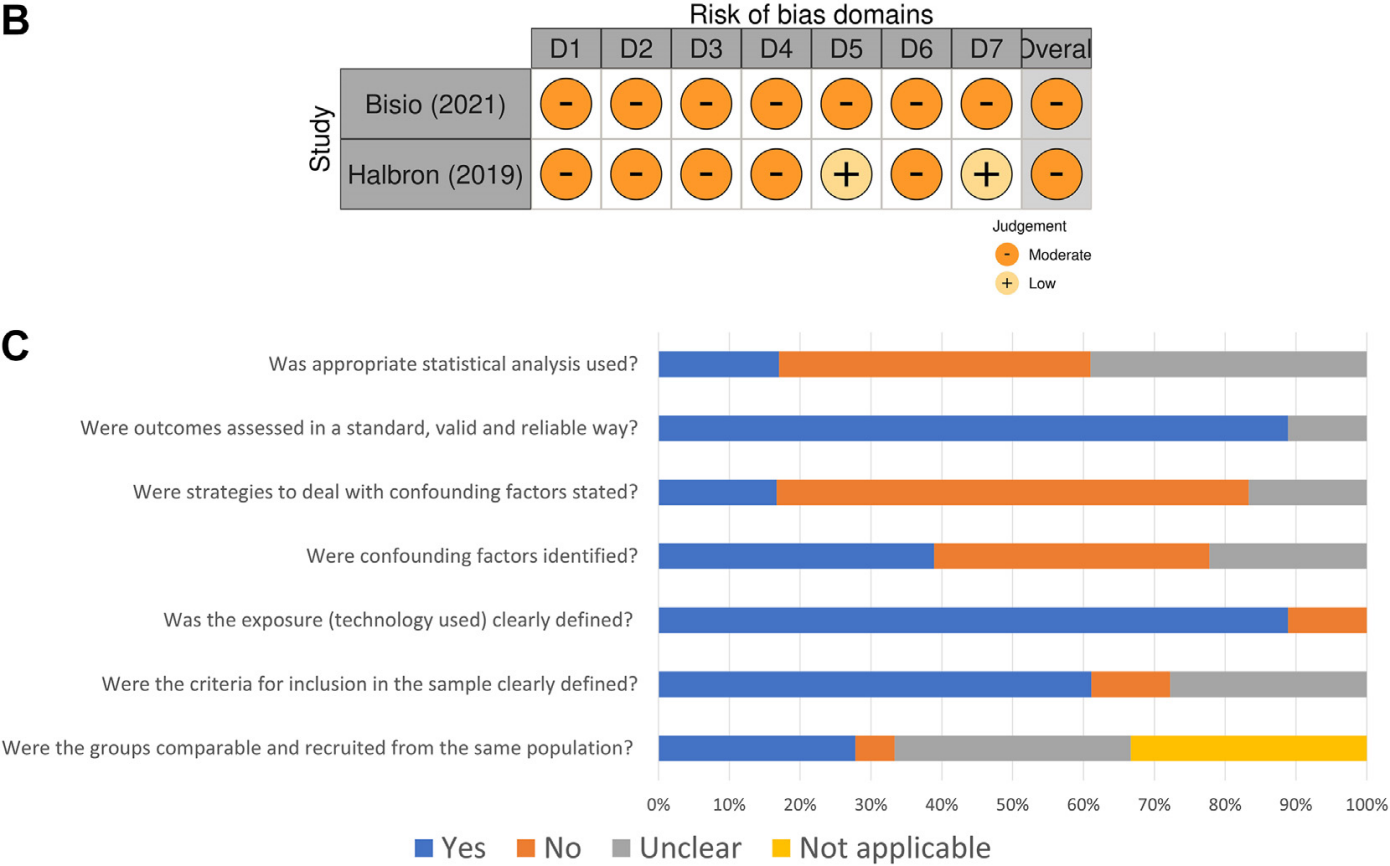
Risk of Bias assessment for randomized trials (A), non-randomized trials (B), and Aggregate Risk of Bias (JBI assessment) for observational studies (C). Legend: Risk of bias Domains. 4A: D1: Bias arising from the randomization process. D1b: bias arising from the timing of identification or recruitment of participants or arising from period and carryover effects. D2: bias due to deviations from the intended interventions. D3: bias due to missing outcome data. D4: bias in measurement of the outcome. D5: bias in selection of the reported result. 4B: D1: Bias due to confounding. D2: Bias in selection of participants into the study. D3: Bias in classification of interventions. D4: bias due to deviations from the intended interventions. D5: bias due to missing data. D6: Bias in measurement of outcomes. D7: Bias in selection of the reported result.

Less than half (n = 10) of the RCTs included in this review were found to have some degree of concern in at least one domain without being at high risk of bias for any^29^^,^^30^^,^^36^, ^37^, ^38^, ^39^^,^^41^^,^^42^^,^^45^^,^^50^ (Fig. 4A) in addition to three that were classified as high risk.^32^^,^^47^^,^^48^ The main source of potential bias was a result of the inability to blind the participants (performance bias) and researchers to the group allocation (observer bias) for all studies. The Risk Of Bias In Non-randomized Studies (ROBINS) tool also shows a moderate risk of bias overall in both studies (Fig. 4B).^51^^,^^52^ Observational studies’ risk of bias was assessed using JBI tools, the main concerns for possible sources of bias were related to lack of strategies to deal with confounding factors (68% of the studies didn’t have a plan and 16% were unclear),^27^^,^^53^^,^^54^^,^^56^^,^^57^^,^^60^, ^61^, ^62^, ^63^, ^64^, ^65^, ^66^, ^67^ additionally, only 21% attempted to adjust for baseline level of FOH or other covariates in their analysis.^26^^,^^28^^,^^55^^,^^59^ The aggregate risk of bias for observational studies is presented in Fig. 4C.

**Fig. 5:**
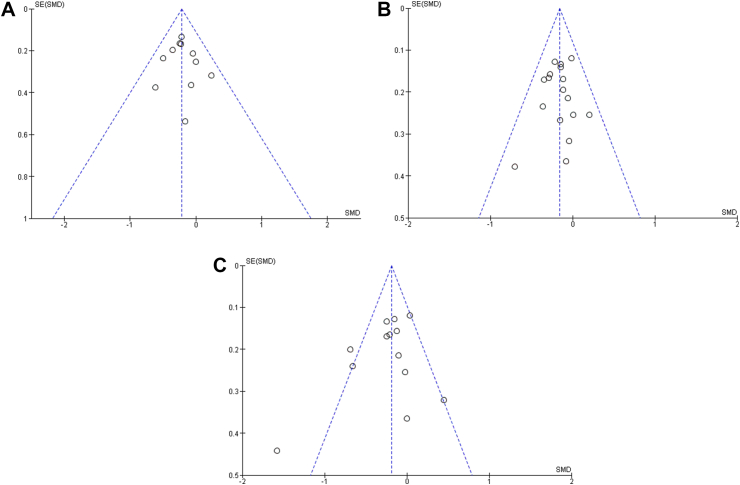
Funnel plots demonstrating no publication bias among the RCT studies in terms of change in FOH using (A) HFS-T, (B) HFS-W, and (C) HFS-B scales. Footnotes: SE, standard error; SMD, standard mean difference.

**Fig. 6:**
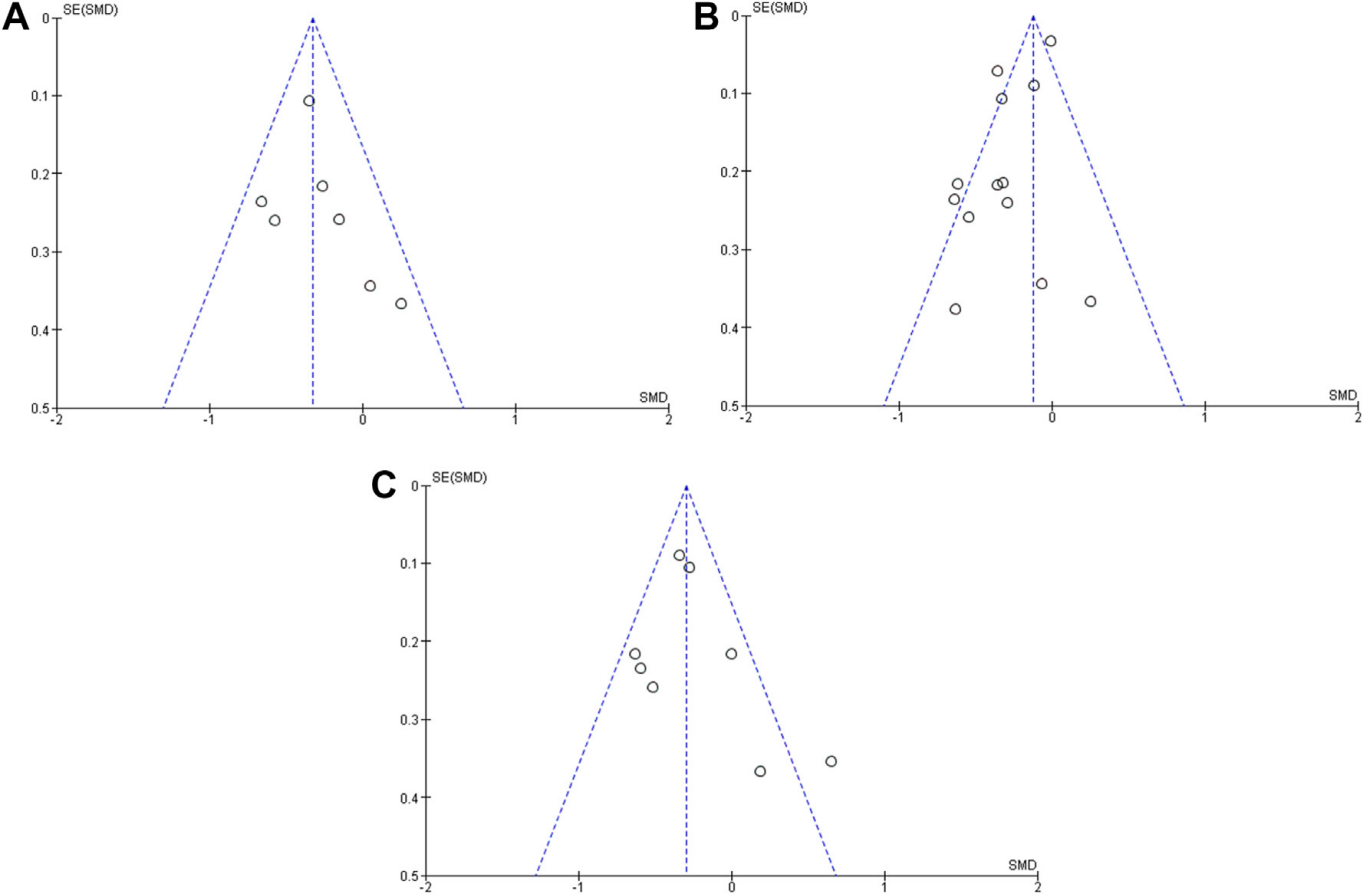
Funnel plots demonstrating no publication bias among the non-RCT studies in terms of change in FOH using (A) HFS-T and (C) HFS-B scales and publication bias among the non-RCT studies in terms of change in FOH using (B) HFS-W scale. Footnotes: SE, standard error; SMD, standard mean difference.

There was no evidence of publication bias in terms of change in FOH using (A) HFS-T, (B) HFS-W, and (C) HFS-B scales in the RCT studies comparing intervention and control methods using Egger’s regression test (p = 0.47, p = 0.42, p = 0.42, respectively). Moreover, a visual inspection of the funnel plot revealed a symmetrical funnel (Fig. 5). Similarly, we found no evidence of publication bias in terms of change in FOH using (A) HFS-T and (C) HFS-B scales in the non-RCT studies comparing intervention and control methods using Egger’s regression test (p = 0.26, p = 0.23, respectively). The visual inspection of the funnel plot revealed a symmetrical funnel. However, we observed evidence of publication bias in terms of change in FOH using (B) HFS-W scale in the non-RCT studies comparing intervention and control methods using Egger’s regression test (p = 0.01), which was confirmed by the asymmetrical funnel plot (Fig. 6).

Leave-one-out sensitivity analyses were performed to further identify the possible source of heterogeneity in the pooled analysis of the SMD of FOH outcome between intervention and control groups (Supplemental Table S1). The results showed no significant difference in outcome, and the meta-analysis had strong reliability.

## Discussion

Findings from this analysis support the hypothesis that using diabetes technologies (specifically; rtCGM, SAP, and AID) can decrease FOH even in the absence of an impact on the frequency of hypoglycaemia. To our knowledge, this is the first meta-analysis of studies exploring the use of different diabetes technologies (including novel AID) that reports on various aspects of FOH (worries and behaviours) while accounting for the impact of these technologies on the frequency of hypoglycaemia. In line with Martyn-Nemeth et al., qualitative review of FOH, this analysis confirms that reducing FOH might not always be dependent on lowering biochemical hypoglycaemia.^10^

Overall, the magnitude of the effects reported for diabetes technologies on HFS-T scores varied from small to moderate. This is consistent with recent findings from a meta-analysis of 11 studies exploring the impact of CGM use on improving emotional well-being.^77^ The meta-analysis also reported a reduction in HFS-W in CGM users compared with CBG users (Cohen d = −0.24 [−0.41 to −0.07]), although there was no discussion of the impact of CGM use on HFS-B nor were the nuances between rtCGM and isCGM discussed.^77^

Our findings show a decrease in the emotional burden related to hypoglycaemia (lower HFS-W scores), specifically with rtCGM use; however, the limited impact on behaviours (HFS-B) should be further explored to better understand the impact of rtCGM on both adaptive and maladaptive behaviours in avoiding hypoglycaemia. It is worth noting that the current way of reporting on the total or subscale scores of the HFS and HFS-II does not allow for differentiation between behaviours related to best practices (such as reducing insulin when feeling low or keeping simple sugars easily accessible) and those that are maladaptive (such as keeping blood glucose purposely high or avoiding physical and social activities to avoid hypoglycaemia). Thus, future studies should explore these items beyond just the aggregate scores to have a clearer picture of the type of fear and associated behaviours in a certain population. Our analysis also explored more novel technologies, such as SAP and AID use and their reported impact on FOH. AID use significantly reduced HFS-T and HFS-W scores in RCT studies, while SAP showed a similar trend in non-RCT studies. These findings show that these technologies can reduce FOH and possibly related compensatory behaviours. The automated suspension of insulin delivery decreases the risk of hypoglycaemia and might offer a sense of safety that extends beyond its impact on the occurrence of hypoglycaemia episodes. Additionally, AID use compared to SAP, CSII, and MDI was associated with a reduction in FOH.^33^^,^^38^^,^^53^^,^^63^ Showcasing evidence of improvement in both previous technology users and technology-naive individuals.

As there is currently no evidence highlighting the optimal time frame of diabetes technology use required for improvement in FOH, we arbitrarily chose seven days of use as a minimum inclusion criterion, as we hypothesized that any duration of wear below seven days would be too brief to allow participants to live with the technology during both weekdays and weekends and thus cover a variety of daily changes. However, all the included studies in this review were four weeks or longer in duration.

The current work shows a promising trend supporting the use of diabetes technologies as a tool to reduce the burden of hypoglycaemia and FOH. However, it is important to highlight some of the current literature limitations. The quality assessment highlighted a series of concerns, although in the majority they were not deemed to be at high risk of bias. Some of the concerns highlighted included either the lack of reporting or adjusting for relevant participants’ baseline characteristics such as baseline levels of FOH, hypoglycaemia history or awareness,^28^^,^^39^^,^^43^^,^^54^, ^55^, ^56^^,^^58^^,^^61^^,^^66^ previous experience with diabetes technologies, diabetes duration,^49^ as well as significant differences at baseline in those characteristics between control and experimental groups. For example, Reddy et al. (2018), Hermanides et al. (2011), and Bosi et al. (2019) reported higher baseline FOH scores in the intervention group. Although the decrease between baseline and end of follow-up was numerically bigger in the intervention group, since this analysis only looked at the end of follow-up, as we did not have access to individual participants' raw scores, that significant decrease compared to controls was not captured in our quantitative analysis.^31^^,^^36^^,^^45^

Specifically, in RCTs, blinding participants to their study allocation proved difficult due to the nature of the intervention which could also be a limitation (performance and observer bias risk). Additionally, the JBI assessment of observational studies showed that the majority of the studies did not use appropriate statistical analysis for this outcome (FOH was not part of the primary analysis, not accounting for baseline covariates, confounding factors were not identified and no strategies to deal with them).

Lack of information on a participant’s hypoglycaemia history and their motivation to use technology can make it challenging to formulate clinically relevant conclusions, as an individual’s glycaemic history can significantly affect their level of FOH and how they respond to technologies. While some studies excluded participants with IAH or a history of severe hypoglycaemia,^30^^,^^42^^,^^44^^,^^54^ the majority included participants who have a history of severe hypoglycaemia and/or IAH.^31^^,^^33^^,^^40^^,^^47^^,^^55^^,^^58^^,^^67^^,^^69^ Additionally, two studies included the reason for using technology as a variable in their analysis and both found that PWT1D whose indication to use technology was hypoglycaemia and individuals with IAH at baseline benefitted more in terms of FOH reduction.^55^, ^69^ While most of the studies reported the level of FOH at baseline, only one study attempted to classify elevated FOH using a cut-off and was the only one to report the extent of elevated FOH in their sample at baseline.^48^ Furthermore, sample size limitations (n ≤ 40) could have made the data underpowered to detect significant changes in reported FOH for the individual studies.^32^^,^^37^^,^^40^^,^^41^^,^^45^^,^^47^^,^^50^, ^51^, ^52^, ^53^, ^54^^,^^63^ Thus, we used Hedges g as it is better adapted for such samples. Additionally, small sample sizes might not accurately represent the diverse T1D population and could have biased the conclusions related to FOH levels and participants’ interaction with the technology. Most of the studies were conducted in Europe and North America, both locations with well-resourced healthcare systems and where diabetes technologies are available, although not always accessible depending on health insurance coverage and costs. The current literature lacks data on patient-outcomes in less well-resourced health-care systems that might not have access to the most novel technologies and therapies and who might in fact benefit from them the most. Additionally, the samples in the included studies were predominantly non-Hispanic white participants of high socioeconomic status, with access to specialized diabetes care, uncomplicated glycaemic management, low FOH at baseline, and high baseline QOL levels.^26^, ^27^, ^28^, ^29^, ^30^, ^31^^,^^33^, ^34^, ^35^, ^36^, ^37^, ^38^, ^39^, ^40^^,^^42^, ^43^, ^44^, ^45^, ^46^, ^47^, ^48^, ^49^^,^^51^, ^52^, ^53^, ^54^, ^55^, ^56^^,^^58^, ^59^, ^60^, ^61^, ^62^^,^^65^^,^^67^^,^^68^ Moreover, sex and gender differences were not explored in any of the studies, yet the current literature suggests that women are more worried about hypoglycaemia than men.^78^^,^^79^ Future studies exploring this question (and similar patient-reported outcome measures) should consider collecting information on participant characteristics, their hypoglycaemia history, their motivation to use technology, and baseline FOH. Such information can facilitate the translation of these findings to draw clinically relevant conclusions.

Overall reducing FOH can have significant clinical implications on PWT1D. While some FOH can be protective as it would warrant some level of attention and preparedness to avoid hypoglycaemic events, elevated or uncontrolled FOH can lead to the development of maladaptive behaviours and unnecessary physical and social activity restrictions^80^ that impact both the quality of life of the individual and their overall diabetes management. In this review, we show that the use of diabetes technologies which provides flexibility and timely feedback, can significantly reduce the burden of FOH in PWT1D and consequently reduce its impact. We also found no evidence that the reduction of FOH is associated with an increase in hypoglycaemia which confirms that we can safely reduce the mental burden of hypoglycaemia without increasing risky hypoglycaemia-inducing behaviours. Other interventions have been proposed to help reduce this burden and could be used in combination with diabetes technology to provide a more patient-centred approach or when technology is not available. For instance, cognitive behavioural therapy-based interventions have shown the potential to decrease FOH-related behaviours.^81^ Training or education-based interventions such as the Blood Glucose Awareness Training have also been shown to decrease FOH in adults.^82^ Current evidence on the benefit of training interventions alone vs technology use is limited,^83^ however, the inclusion of these interventions alongside diabetes technologies may provide further benefits and provide PWT1D additional tools to significantly decrease FOH and improve T1D management.

To our knowledge, this is the most complete review assessing the impact of a variety of diabetes technologies on FOH in PWT1D. This review found pertinent evidence supporting a role for rtCGM, SAP, and AID technologies, and to a lesser extent CSII technologies, in reducing FOH when used for at least four weeks. This decrease in FOH with diabetes technology use was independent of the reduction in hypoglycaemia frequency, further confirming a specific benefit for reducing FOH. Future studies exploring the real-world impact of diabetes technologies on FOH should be designed with FOH as the primary outcome, use a validated tool to measure FOH, and adjust accordingly when evaluating technology use as FOH-related worries and behaviours could be a highly impactful factor to improve overall diabetes management.

## Contributors

M.K.T designed the study and A-SB, TMP, and J-FY. provided supervision and contributed to study design and conception. MKT planned and conducted the statistical analysis with assistance from a biostatistician. M.K.T. and A.K. ran the literature search, assessed study eligibility, data extraction, and assessed study quality and risk of bias with assistance from L.H.

M.K.T. and A.K. wrote the first draft of the manuscript. MKT, AK, LH, TP, JFY, and ASB contributed to the interpretation and subsequent edits of the manuscript. All authors read and approved the final manuscript. MKT., AK, LH, and ASB have accessed and verified the underlying data.

## Data sharing statement

All data are available in the referenced articles as listed in the References or by contacting their respective corresponding authors.

## Declaration of interests

M.T., A.K., L.H., and T.P. have no conflicts of interest to disclose.

A.S.B. reports the following: **Research** grants or awards: Fonds de recherche du Québec en Santé Research, Canadian Institutes of Health Research, Juvenile Diabetes Research Foundation, Diabète Québec, and **speaker** fees: Dexcom, J.F.Y., reports the following: **Honoraria:** Novo Nordisk, Eli Lilly, Sanofi, Abbott, Dexcom, Bayer.
