# Supplementary material for: Effect of diabetes technologies on the fear of hypoglycaemia among people living with type 1 diabetes: a systematic review and meta-analysis
Source: eClinicalMedicine. 2023 Aug 4;62:102119. doi: 10.1016/j.eclinm.2023.102119 (PMC10430205; doi:10.1016/j.eclinm.2023.102119)
Supplement: Supplementary Materials [file mmc1.pdf]

## Table of Contents

Supplemental material 1: Search strategy ........ 2

Supplemental table 1· Leave-one-out sensitivity analysis of SMD of the hypoglycemia fear survey scores ........ 5

Supplemental table 2: Risk of Bias assessment (JBI assessment) for observational studies ........ 7

Supplemental table 3: Excluded studies and reason for their exclusion ........ 8

Supplemental figure 1: RCT meta-analysis results - hypoglycemia decrease subgroup analysis (Less hypoglycemia, no hypoglycemia information (NI), or no difference (ND)): ........ 9

Total HFS score ........ 10

HFS-Behaviour score ........ 11

HFS-Worry score ........ 12

Supplemental figure 2: nonRCT meta-analysis results – hypoglycemia decrease subgroup analysis (Less hypoglycemia, no hypoglycemia information (NI), or no difference (ND)) ........ 13

Total HFS score ........ 14

HFS-Behaviour score ........ 15

HFS-Worry score ........ 16

Supplemental figure 3: RCT meta-analysis results – subgroup analysis by the duration of technology use ........ 17

Total HFS score ........ 18

HFS-Behaviour score ........ 19

HFS-Worry score ........ 20

Supplemental figure 4: nonRCT meta-analysis results – subgroup analysis by the duration of technology use ........ 21

Total HFS score ........ 22

HFS-Behaviour score ........ 23

HFS-Worry score ........ 24

**Appendix 1: Search strategy**

**Search strategies:**

PubMed, MEDLINE, Web of Science and Scopus

Published between 2000 to May 21st, 2023

English OR French

No further restrictions to avoid unnecessarily limiting the search (goal to get as complete of a search as possible and catch all articles)

**PubMed Search Strategy**

Search: ((Fear of hypoglycemia[Text Word]) OR (FOH[Text Word]) OR (Distress[Text Word]) OR (Patient-reported outcomes[Text Word]) OR (patient experience[Text Word]) OR (Worr*[Text Word]) OR (Anxiety[Text Word]) OR (Behaviour[Text Word]) OR (Behavior[Text Word]) OR (Health related quality of life[Text Word]) OR (well-being[Text Word]) OR
(Psychosocial outcome*[Text Word]) OR (fear [MeSH]) OR (Behavior and Behavior Mechanisms [MeSH]) OR (Quality of Life[MeSH])) AND ((Hypoglycemi*[Text Word]) OR (Hypoglycaemi*[Text Word]) OR (Hypoglycemia[MeSH])) AND ((Autoimmune diabetes[Text Word]) OR (juvenile diabetes[Text Word]) OR (Insulin-Dependent diabetes [Text Word]) OR (IDDM [Text Word]) OR (diabetes mellitus type 1[Text Word]) OR (diabetes mellitus, type 1[MeSH])) AND ((Insulin Infusion System* [Text Word]) OR (continuous subcutaneous insulin infusion [Text Word]) OR (continuous subcutaneous injection [Text Word]) OR (CSII [Text Word])OR (pump therapy [Text Word]) OR (Artificial pancreas [Text Word]) OR (Pancreas, Artificial[Mesh]) Or (Closed loop control [Text Word]) Or (insulin infusion [Text Word]) OR (insulin pump [Text Word])OR (automated insulin delivery [Text Word]) OR (sensor augmented pump therapy [Text Word]) OR (continuous glucose monitor* [Text Word]) OR (glucose monitor* [Text Word]) OR (flash glucose monitor* [Text Word]) OR (Freestyle libre [Text Word]) OR (Intermittently scanned continuous glucose monitor* [Text Word]) OR ( Real time continuous glucose monitor* [Text Word]) OR (Insulin Infusion Systems [Mesh]) OR (Blood Glucose Self-Monitoring [MeSH]) OR ( FGM [Text Word]) OR (CGM [Text Word]) OR (diabetes technolog*[Text Word]) OR (technolog* [Text Word]) OR (Biomedical technology [MeSH]) OR (technology advances [Text Word])) Filters: from 2000 - 2023

**Web of Science**

1· TS = Fear of hypoglycemia OR TS = FOH OR TS = Distress OR TS = Patient-reported outcome* OR TS = patient experience OR TS = Worr* OR TS = Anxiety OR TS = Behaviour OR TS = Behavior OR TS = Health related quality of life OR TS = well-being OR TS = Psychosocial outcome* OR TS = Fear* OR TS = Behavior OR TS = Quality of Life

2· TS = Hypoglycemi* OR TS = Hypoglycaemi*

3·  TS = Autoimmune diabetes OR TS = juvenile diabetes OR TS = Insulin-Dependent diabetes OR TS = IDDM OR TS = diabetes mellitus type 1

4·  TS = Insulin Infusion System* OR TS = continuous subcutaneous infusion OR TS = continuous subcutaneous insulin infusion OR TS = continuous subcutaneous injection OR TS = CSII OR TS = CGM OR TS = FGM OR TS = pump therapy OR TS = Artificial pancreas OR TS = Closed loop OR TS = automated insulin delivery OR TS = sensor augmented pump therapy OR TS = continuous glucose monitor* OR TS = flash glucose monitor* OR TS = Freestyle libre OR TS = Intermittently scanned continuous glucose monitor* OR TS = Real time continuous glucose monitor* OR TS = Blood Glucose *Monitor* OR TS = diabetes technolog* OR TS = Biomedical technolog* OR TS = technology advance* Or TS = insulin infusion OR TS = insulin pump OR TS = glucose monitor*

5·  #1 AND #2 AND #3 AND #4

**Medline via Ovid**

1. Fear of hypoglycemia·mp· or Patient-reported outcome*·mp· or patient experience·mp· or worry·mp· or anxiety·mp· or behavio*r·mp· or Health related quality of life·mp· or FOH·mp· or fear·mp· or distress·mp· or Psychosocial outcome·mp· or "Quality of Life"/ or Patient-Centered Care/ or Patient Reported Outcome Measures/ or Fear/ or Psychological Distress/ or Anxiety/ or Behavior/
2. Hypoglycaemi*·mp· or Hypoglycemi*·mp· or Hypoglycemia/
3. Diabetes Mellitus, Type 1/ or Diabetes Mellitus, Type 1·mp· or Autoimmune diabetes·mp· or juvenile diabetes·mp· or Insulin-Dependent diabetes·mp· or IDDM·mp·
4. Insulin Infusion System*·mp· or continuous subcutaneous infusion·mp· or continuous subcutaneous insulin infusion·mp· or continuous subcutaneous injection·mp· or csii·mp· or fgm·mp· or cgm·mp· or pump therapy·mp· or Artificial pancreas·mp· or Closed loop·mp· or automated insulin delivery·mp· or sensor augmented pump therapy·mp· or continuous glucose monitor*·mp· or flash glucose monitor*·mp· or Freestyle libre·mp· or Intermittently scanned continuous glucose monitor*·mp· or Real time continuous glucose monitor*·mp· or diabetes technology·mp· or Biomedical technology·mp· or technology advance*·mp· or insulin infusion·mp· or insulin pump·mp· or glucose monitor*·mp· or Insulin Infusion Systems/ or Monitoring, Ambulatory/ or Blood Glucose/ or Blood Glucose Self-Monitoring/ or Pancreas, Artificial/ or Biomedical Technology/
5. 1 and 2 and 3 and 4
6. limit 5 to yr="2000 -Current"

**Scopus**

( "Fear of hypoglycemia" OR foh OR distress OR "Patient-reported outcome*" OR "patient experience*" OR worr* OR anxiety OR behaviour OR behavior OR "Health related quality of life" OR "well-being" OR "Psychosocial outcome*" OR fear* OR behavior OR "Quality of Life" ) AND ( hypoglycemi* OR hypoglycaemi* ) AND ( "Autoimmune diabetes" OR "juvenile diabetes" OR "Insulin-Dependent diabetes" OR "IDDM" OR "diabetes mellitus type 1" ) AND ( "Insulin Infusion System*" OR "continuous subcutaneous infusion" OR "continuous subcutaneous insulin infusion" OR "continuous subcutaneous injection" OR csii OR cgm OR fgm OR "pump therapy" OR "Artificial pancreas" OR "Closed loop" OR "automated insulin delivery" OR " pump therapy" OR "continuous glucose monitor*" OR "flash glucose monitor*" OR "Freestyle libre" OR "Intermittently scanned continuous glucose monitor*" OR "Real time continuous glucose monitor*" OR "Blood Glucose *Monitor*" OR "diabetes technolog*" OR "Biomedical technolog*" OR "technology advance*" OR "insulin infusion" OR "insulin pump" OR "glucose monitor*" ) AND ( LIMIT-TO ( PUBYEAR , 2023 ) OR LIMIT-TO ( PUBYEAR , 2022 ) OR LIMIT-TO ( PUBYEAR , 2021) OR LIMIT-TO ( PUBYEAR , 2020 ) OR LIMIT-TO ( PUBYEAR , 2019 ) OR LIMIT-TO ( PUBYEAR , 2018 ) OR LIMIT-TO ( PUBYEAR , 2017 ) OR LIMIT-TO ( PUBYEAR , 2016 ) OR LIMIT-TO ( PUBYEAR, 2015 ) OR LIMIT-TO ( PUBYEAR , 2014 ) OR LIMIT-TO ( PUBYEAR , 2013 ) OR LIMIT-TO ( PUBYEAR , 2012 ) OR LIMIT-TO ( PUBYEAR , 2011 ) OR LIMIT-TO ( PUBYEAR , 2010 ) OR LIMIT-TO ( PUBYEAR , 2009 ) OR LIMIT-TO ( PUBYEAR, 2008 ) OR LIMIT-TO ( PUBYEAR , 2007 ) OR LIMIT-TO ( PUBYEAR , 2006 ) OR LIMIT-TO ( PUBYEAR , 2005 ) OR LIMIT-TO ( PUBYEAR , 2004 ) OR LIMIT-TO ( PUBYEAR , 2003 ) OR LIMIT-TO ( PUBYEAR , 2002 ) OR LIMIT-TO ( PUBYEAR , 2001 ) OR LIMIT-TO ( PUBYEAR , 2000 ) )

**Inclusion/exclusion criteria**

**Articles excluded for not meeting the stated eligibility criteria (n=)**

No assessment of fear OR use of non-validated tools (n=)

Short (less than 7 days) duration of follow-up (n=)

Not assessing use of a new technology (n=)

Assessing use of smartphone application/website intervention (n=)

Not focused on Type 1 Diabetes (n = )

Pregnancy, critical care setting OR Intraperitoneal insulin infusion systems (n = )

Intervention combinations (technology +/- telehealth OR psychological intervention) (n=)

Commentaries, review articles, consensus guidelines, OR protocols (n = )

**Supplemental table 1· Leave-one-out sensitivity analysis of SMD of the hypoglycemia fear survey scores**

| Study design | Scale | Excluded article | SMD [95% CI] | P value |
|---|---|---|---|---|
| RCT | HFS-total | Beck et al 2010 | -0.185 [-0.312; -0.058] | <0.01 |
| | | Bosi et al 2019 | -0.183 [-0.303; -0.062] | <0.01 |
| | | Burckhard et al 2021 | -0.185 [-0.297; -0.074] | <0.01 |
| | | Choudhary et al 2022 | -0.168 [-0.268; -0.069] | <0.01 |
| | | Heinemann et al 2018 | -0.186 [-0.307; -0.065] | <0.01 |
| | | Kropff et al 2017 | -0.204 [-0.314; -0.093] | <0.01 |
| | | Kudva et al 2021 | -0.174 [-0.286; -0.061] | <0.01 |
| | | Little et al 2014 (a) | -0.205 [-0.318; -0.092] | <0.01 |
| | | Little et al 2014 (b) | -0.209 [-0.319; -0.099] | <0.01 |
| | | McAuley et al 2022 | -0.195 [-0.308; -0.081] | <0.01 |
| | | Reddy et al 2018 | -0.208 [-0.306; -0.111] | <0.01 |
| | | Thomas et al. 2007 | -0.192 [-0.305; -0.078] | <0.01 |
| | HFS-W | Beck et al 2010 | -0.148 [-0.215; -0.080] | <0.01 |
| | | Bosi et al. 2019 | -0.142 [-0.207; -0.076] | <0.01 |
| | | Burckhard et al. 2021 | -0.143 [-0.206; -0.081] | <0.01 |
| | | Choudhary et al 2022 | -0.140 [-0.201; -0.078] | <0.01 |
| | | Heinemann et al 2018 | -0.149 [-0.216; -0.083] | <0.01 |
| | | Hermanides et al. 2011 | -0.157 [-0.214; -0.101] | <0.01 |
| | | Kropff et al. 2017 | -0.152 [-0.215; -0.089] | <0.01 |
| | | Kudva et al. 2021 | -0.149 [-0.215; -0.083] | <0.01 |
| | | Lind et al, 2017 | -0.167 [-0.229; -0.105] | <0.01 |
| | | Little et al, 2014 (a) | -0.151 [-0.216; -0.086] | <0.01 |
| | | Little et al, 2014 (b) | -0.151 [-0.216; -0.086] | <0.01 |
| | | McAuley et al. 2022 | -0.148 [-0.213; -0.084] | <0.01 |
| | | Oskarsson et al, 2018 | -0.137 [-0.201; -0.073] | <0.01 |
| | | Polonsky et al. 2017 | -0.134 [-0.194; -0.074] | <0.01 |
| | | Pratley et al. 2020 | -0.148 [-0.215; -0.081] | <0.01 |
| | | Reddy et al. 2018 | -0.149 [-0.213; -0.085] | <0.01 |
| | | Visser et al. 2021 | -0.139 [-0.205; -0.072] | <0.01 |
| | | Wheeler et al. 2022 | -0.147 [-0.212; -0.082] | <0.01 |
| | HFS-B | Beck et al 2010 | -0.175 [-0.326; -0.023] | 0.03 |
| | | Bosi et al. 2019 | -0.179 [-0.329; -0.029] | 0.02 |
| | | Choudhary et al 2022 | -0.155 [-0.278; -0.032] | 0.02 |
| | | Heinemann et al 2018 | -0.176 [-0.325; -0.027] | 0.02 |
| | | Kropff et al. 2017 | -0.189 [-0.332; -0.045] | 0.01 |
| | | Kudva et al. 2021 | -0.142 [-0.251; -0.033] | 0.02 |
| | | Lind et al, 2017 | -0.213 [-0.344; -0.083] | < 0.01 |
| | | Little et al, 2014 (a) | -0.187 [-0.333; -0.041] | 0.02 |
| | | Little et al, 2014 (b) | -0.187 [-0.334; -0.041] | 0.02 |
| | | McAuley et al. 2022 | -0.186 [-0.328; -0.044] | 0.01 |
| | | Oskarsson et al, 2018 | -0.188 [-0.338; -0.038] | 0.02 |
| | | Reddy et al 2018 | -0.198 [-0.322; -0.074] | < 0.01 |
| | | Visser et al, 2021 | -0.187 [-0.340; -0.034] | 0.02 |


5

| | | | | |
|---|---|---|---|---|
| | | Wheeler et al, 2022 | -0.176 [-0.320; -0.032] | 0.02 |
| Non-RCT | HFS-total | Bisio et al, 2021 | -0.350 [-0.541, -0.160] | < 0.01 |
| | | Boscari et al, 2022 (a) | -0.286 [-0.490, -0.082] | 0.02 |
| | | Boscari et al, 2022 (b) | -0.302 [-0.528, -0.075] | 0.02 |
| | | Halbron et al, 2019 | -0.344 [-0.561, -0.127] | < 0.01 |
| | | Murata et al, 2021 | -0.334 [-0.581, -0.087] | 0.02 |
| | | Norgaard et al 2013 | -0.288 [-0.629, 0.052] | 0.08 |
| | | Perez Garcia et al 2015 | -0.341 [-0.576, -0.075] | 0.01 |
| | HFS-W | Bisio et al. 2021 | -0.268 [-0.410; -0.126] | <0.01 |
| | | Boscari et al 2022 (a) | -0.295 [-0.429; -0.160] | <0.01 |
| | | Boscari et al. 2022 (b) | -0.264 [-0.408; -0.121] | <0.01 |
| | | Charleer et al. 2020 | -0.253 [-0.390; -0.116] | <0.01 |
| | | Charleer et al. 2018 | -0.287 [-0.433; -0.141] | <0.01 |
| | | Halbron et al. 2019 | -0.319 [-0.437; -0.201] | <0.01 |
| | | Murata et al 2021 | -0.305 [-0.456; -0.155] | <0.01 |
| | | Nefs et al. 2019 | -0.269 [-0.426; -0.113] | <0.01 |
| | | Norgaard et al. | -0.278 [-0.429; -0.127] | <0.01 |
| | | Rouhard et al. 2020 | -0.254 [-0.391; -0.118] | <0.01 |
| | | Shaban et al. 2017 | -0.280 [-0.431; -0.130] | <0.01 |
| | | Wu et al. 2020 | -0.277 [-0.433; -0.121] | <0.01 |
| | HFS-B | Bisio et al. 2021 | -0.298 [-0.619; 0.022] | 0.06 |
| | | Boscari et al 2022 (a) | -0.216 [-0.563; 0.131] | 0.18 |
| | | Boscari et al. 2022 (b) | -0.223 [-0.587; 0.141] | 0.18 |
| | | Halbron et al. 2019 | -0.326 [-0.494; -0.158] | < 0.01 |
| | | Murata et al 2021 | -0.323 [-0.544; -0.102] | 0.01 |
| | | Norgaard et al. | -0.240 [-0.642; 0.162] | 0.19 |
| | | Rouhard et al. 2020 | -0.227 [-0.627; 0.173] | 0.21 |
| | | Shaban et al. 2017 | -0.216 [-0.543; 0.111] | 0.16 |


6

**Supplemental table 2: Risk of Bias assessment (JBI assessment) for observational studies**

| Study ID | Were the groups comparable and recruited from the same population? | Were the criteria for inclusion in the sample clearly defined? | Was the exposure (Technology used) clearly defined? | Were confounding factors identified? | Were strategies to deal with confounding factors stated? | Were outcomes assessed in a standard, valid and reliable way? | Was appropriate statistical analysis used? |
|---|---|---|---|---|---|---|---|
| Barnard et al 2008 | Not applicable | Unclear | Yes | Yes | Yes | Yes | No |
| Boscari et al 2022 | Yes | Yes | Yes | No | No | Yes | No |
| Boscari et al 2022 | Yes | Yes | Yes | No | No | Yes | No |
| Boulet et al 2016 | Not applicable | Unclear | Yes | Yes | No | Yes | Yes |
| Charleer et al 2020 | Yes | Yes | Yes | No | No | Yes | No |
| Charleer et al 2018 | Unclear | No | Yes | Unclear | Unclear | Yes | Yes |
| Linkeschova et al 2002 | Unclear | Unclear | No | Unclear | No | Yes | Unclear |
| McAuley et al 2021 | Not applicable | Yes | Yes | Yes | No | Yes | No |
| Munshi et al 2022 | Not applicable | Yes | Yes | Yes | No | Yes | No |
| Murata et al 2021 | Unclear | Yes | Yes | Yes | No | Yes | No |
| Nefs et al 2019 | Yes | Unclear | Yes | No | Unclear | Yes | Unclear |
| Nicolucci et al 2008 | Unclear | Yes | Yes | Yes | Yes | Yes | Yes |
| Norgaard et al 2013 | Unclear | Yes | Yes | Yes | Yes | Yes | Unclear |
| Perez-Garcia et al 2015 | Unclear | Yes | Yes | Unclear | No | Yes | Unclear |
| Rouhard et al 2020 | Not applicable | Unclear | Yes | No | No | Yes | Unclear |
| Scheidegger et al 2007 | Not applicable | Yes | Yes | Unclear | Unclear | Unclear | Unclear |
| Shaban et al 2017 | Yes | No | No | No | No | Yes | Unclear |
| Wu et al 2020 | No | Yes | Yes | No | No | Unclear | No |

**Supplemental table 3: Excluded studies and reason for their exclusion**

| **Study** | **Reason for exclusion** |
|---|---|
| Aleppo, G., et al. (2017). "REPLACE-BG: A randomized trial comparing continuous glucose monitoring with and without routine blood glucose monitoring in adults with well-controlled type 1 diabetes." Diabetes Care 40(4): 538-545. | The intervention studied was not not comparing CBG to CGM but the "addition of CBG to CGM". |
| Beato-Vibora, P. I., et al. (2021). "Amelioration of user experiences and glycaemic outcomes with an Advanced Hybrid Closed Loop System in a real-world clinical setting." Diabetes Research and Clinical Practice 178. | Analysis combined adults and pediatric data. |
| Beato -Víbora, P. I., et al. (2020). "Prospective Analysis of the Impact of Commercialized Hybrid Closed-Loop System on Glycemic Control, Glycemic Variability, and Patient-Related Outcomes in Children and Adults: A Focus on Superiority Over Predictive Low-Glucose Suspend Technology." Diabetes Technol Ther 22(12): 912-919. | Analysis combined adults and pediatric data. |
| Brown, S. A., et al. (2017). "Overnight closed-loop control improves glycemic control in a multicenter study of adults with type 1 diabetes." Journal of Clinical Endocrinology and Metabolism 102(10): 3674-3682. | Technology used for less than 7 days (short duration of use of 5 days) |
| Chamberlain, J. J., et al. (2015). "Impact of Frequent and Persistent Use of Continuous Glucose Monitoring (CGM) on Hypoglycemia Fear, Frequency of Emergency Medical Treatment, and SMBG Frequency After One Year." Journal of Diabetes Science & Technology 10(2): 383-388. | The comparison was the frequency of use of the technology, it was not comparing CBG to CGM |
| Davey, R. J., et al. (2012). "The effect of short-term use of the Guardian RT continuous glucose monitoring system on fear of hypoglycaemia in patients with type 1 diabetes mellitus." Primary Care Diabetes 6(1): 35-39. | Technology used for less than 7 days (short duration of use of 3 days) |
| Markowitz, J. T., et al. (2012). "Psychosocial correlates of continuous glucose monitoring use in youth and adults with type 1 diabetes and parents of youth." Diabetes Technol Ther 14(6): 523-526 | Analysis combined both isCGM and rtCGM as one technology. |
| Messer, L. H., et al. (2021). "Basal-IQ technology in the real world: satisfaction and reduction of diabetes burden in individuals with type 1 diabetes." Diabetic Medicine 38(6): e14381. | Analysis included adults and care-givers data. |
| Walker, T. C. and C. B. Yucha (2014). "Continuous glucose monitors: use of waveform versus glycemic values in the improvements of glucose control, quality of life, and fear of hypoglycemia." Journal of Diabetes Science & Technology 8(3): 488-493. | Study compared "blinded CGM" to "unblinded CGM" |

**Supplemental figure 1:** RCT meta-analysis results – hypoglycemia decrease subgroup analysis (Less hypoglycemia, no hypoglycemia information (NI), or no difference (ND)) (A- HFS T score, B- HFS-Behaviour score, and C- HFS-Worry score)

| Study | Experimental Total | Experimental Mean | Experimental SD | Technology | Control Total | Control Mean | Control SD | Control | SMD | 95%−CI | Weight |
|---|---|---|---|---|---|---|---|---|---|---|---|
| **Less** | | | | | | | | | | | |
| Reddy (2018) | 20 | 49.50 | 34.0700 | rtCGM | 20 | 42.00 | 27.4070 | isCGM | 0.24 | [−0.38; 0.86] | 3.8% |
| Heinemann (2018) | 75 | 37.00 | 20.0000 | rtCGM | 66 | 42.20 | 25.6000 | CBG | −0.23 | [−0.56; 0.10] | 13.5% |
| Bosi (2019) | 75 | 33.70 | 19.0000 | SAP | 72 | 38.90 | 23.4000 | CSII | −0.24 | [−0.57; 0.08] | 14.1% |
| Burckhard (2021) | 15 | 51.33 | 28.2200 | AID | 15 | 62.00 | 24.1300 | CSII | −0.40 | [−1.12; 0.33] | 2.8% |
| McAuley (2022) | 15 | 7.17 | 4.9000 | AID | 15 | 7.50 | 4.0900 | SAP | −0.07 | [−0.79; 0.64] | 2.9% |
| **Random effects model** | **200** | | | | **188** | | | | **−0.19** | **[−0.40; 0.03]** | **37.2%** |
| Heterogeneity: $I^2 = 0\%$, $\tau^2 = 0$ | | | | | | | | | | | |
| **ND** | | | | | | | | | | | |
| Thomas (2007) | 7 | 64.00 | 16.0000 | CSII | 7 | 67.00 | 19.0000 | MDI | −0.16 | [−1.21; 0.89] | 1.4% |
| Little (2014) (a) | 41 | 44.00 | 23.0000 | CSII | 46 | 45.00 | 25.0000 | MDI | −0.04 | [−0.46; 0.38] | 8.4% |
| Little (2014) (b) | 45 | 45.00 | 25.0000 | rtCGM | 41 | 45.00 | 24.0000 | CBG | 0.00 | [−0.42; 0.42] | 8.3% |
| Choudhary (2022) | 35 | 35.70 | 23.3800 | AID | 39 | 47.40 | 22.8800 | MDI | −0.50 | [−0.96; −0.04] | 6.9% |
| **Random effects model** | **128** | | | | **133** | | | | **−0.16** | **[−0.56; 0.23]** | **25.0%** |
| Heterogeneity: $I^2 = 0\%$, $\tau^2 = 0.0122$ | | | | | | | | | | | |
| **NI** | | | | | | | | | | | |
| Beck (2010) | 120 | 33.30 | 11.5000 | rtCGM | 106 | 36.00 | 13.6000 | CBG | −0.21 | [−0.48; 0.05] | 21.7% |
| Kropff (2017) | 31 | 23.50 | 16.7000 | AID | 31 | 23.50 | 16.6000 | SAP | 0.00 | [−0.50; 0.50] | 6.0% |
| Kudva (2021) | 81 | 33.00 | 12.0000 | AID | 39 | 38.00 | 18.0000 | SAP | −0.35 | [−0.73; 0.04] | 10.1% |
| **Random effects model** | **232** | | | | **176** | | | | **−0.22** | **[−0.55; 0.12]** | **37.8%** |
| Heterogeneity: $I^2 = 0\%$, $\tau^2 = 0$ | | | | | | | | | | | |
| **Random effects model** | **560** | | | | **497** | | | | **−0.19** | **[−0.30; −0.08]** | **100.0%** |

Heterogeneity: $I^2 = 0\%$, $\tau^2 = 0$
Test for subgroup differences: $\chi^2_2 = 0.15$, df = 2 ($p$ = 0.93)

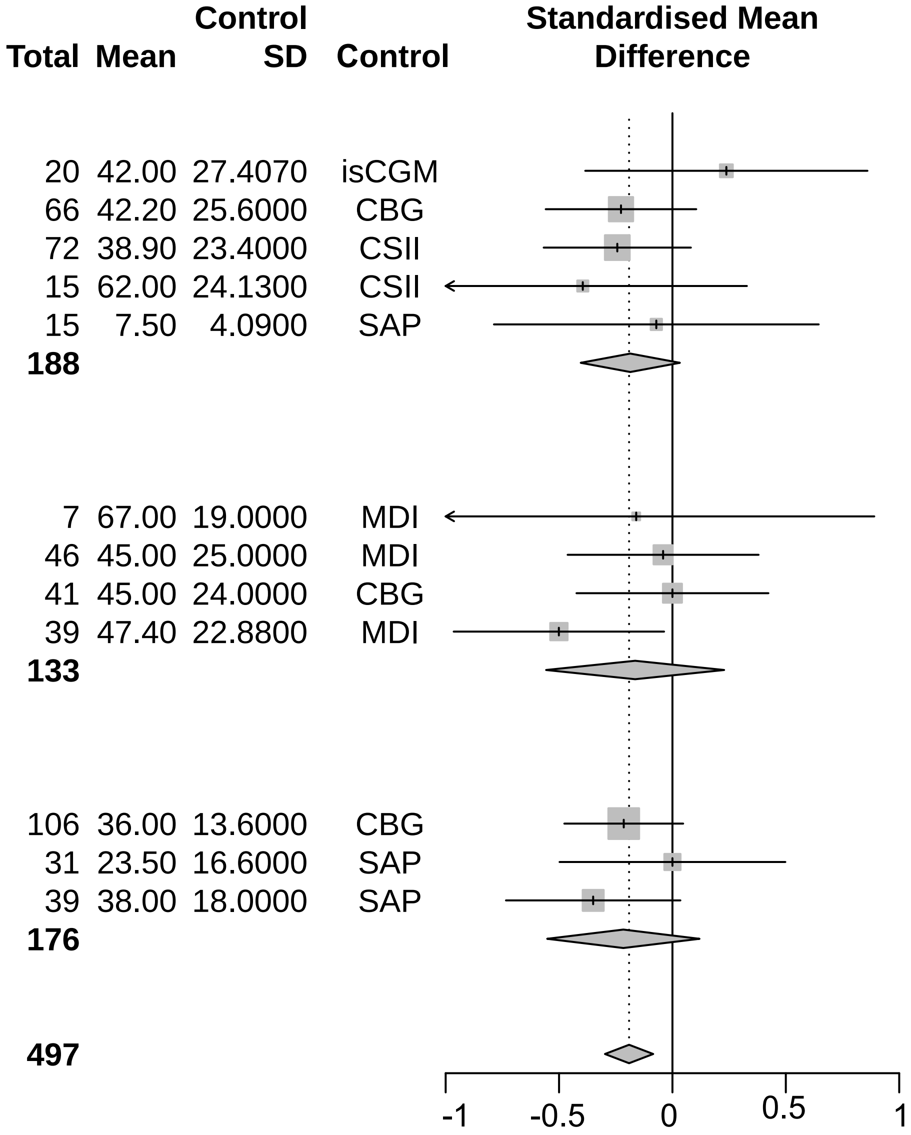


**A-** Use of technology and HFS T score (RCT): Change in hypoglycemia

| Study | Experimental Total | Experimental Mean | Experimental SD | Technology | Control Total | Control Mean | Control SD | Control | SMD | 95%−CI | Weight |
|---|---|---|---|---|---|---|---|---|---|---|---|
| **Less** | | | | | | | | | | | |
| Lind (2017) | 140 | 1.93 | 0.6000 | rtCGM | 140 | 1.91 | 0.5700 | CBG | 0.03 | [−0.20; 0.27] | 13.2% |
| Reddy (2018) | 20 | 20.00 | 11.4800 | rtCGM | 20 | 15.00 | 10.3700 | isCGM | 0.45 | [−0.18; 1.08] | 2.9% |
| Heinemann (2018) | 75 | 14.00 | 7.2593 | rtCGM | 66 | 16.00 | 8.8889 | CBG | −0.25 | [−0.58; 0.09] | 8.4% |
| Oskarsson (2018) | 82 | 13.40 | 5.6000 | isCGM | 81 | 14.20 | 7.3000 | CBG | −0.12 | [−0.43; 0.18] | 9.4% |
| Bosi (2019) | 75 | 15.50 | 8.7000 | SAP | 72 | 17.50 | 10.0000 | CSII | −0.21 | [−0.54; 0.11] | 8.7% |
| Visser (2021) | 124 | 19.00 | 5.4000 | rtCGM | 122 | 19.80 | 5.3500 | isCGM | −0.15 | [−0.40; 0.10] | 12.2% |
| McAuley (2022) | 15 | 2.33 | 2.4500 | AID | 15 | 2.33 | 2.4500 | SAP | 0.00 | [−0.72; 0.72] | 2.3% |
| **Random effects model** | **531** | | | | **516** | | | | **−0.09** | **[−0.24; 0.06]** | **57.0%** |
| Heterogeneity: $I^2$ = 0%, $\tau^2$ = < 0.0001 | | | | | | | | | | | |
| **ND** | | | | | | | | | | | |
| Little (2014) (a) | 41 | 20.00 | 10.0000 | CSII | 46 | 21.00 | 10.0000 | MDI | −0.10 | [−0.52; 0.32] | 5.8% |
| Little (2014) (b) | 45 | 20.00 | 11.0000 | rtCGM | 41 | 21.00 | 10.0000 | CBG | −0.09 | [−0.52; 0.33] | 5.8% |
| Wheeler (2022) | 28 | 16.95 | 6.3000 | AID | 28 | 18.90 | 6.3000 | SAP | −0.31 | [−0.83; 0.22] | 4.0% |
| Choudhary (2022) | 35 | 15.80 | 9.4700 | AID | 39 | 21.80 | 8.5900 | MDI+isCGM | −0.66 | [−1.13; −0.19] | 4.9% |
| **Random effects model** | **149** | | | | **154** | | | | **−0.27** | **[−0.70; 0.15]** | **20.5%** |
| Heterogeneity: $I^2$ = 24%, $\tau^2$ = 0.0181 | | | | | | | | | | | |
| **NI** | | | | | | | | | | | |
| Beck (2010) | 120 | 43.80 | 11.2000 | rtCGM | 106 | 46.80 | 13.3000 | CBG | −0.24 | [−0.51; 0.02] | 11.5% |
| Kropff (2017) | 31 | 12.20 | 8.1000 | AID | 31 | 12.40 | 7.8000 | SAP | −0.02 | [−0.52; 0.47] | 4.4% |
| Kudva (2021) | 81 | 43.00 | 12.0000 | AID | 39 | 52.00 | 15.0000 | SAP | −0.69 | [−1.08; −0.29] | 6.5% |
| **Random effects model** | **232** | | | | **176** | | | | **−0.33** | **[−1.12; 0.46]** | **22.5%** |
| Heterogeneity: $I^2$ = 60%, $\tau^2$ = 0.0581 | | | | | | | | | | | |
| **Random effects model** | **912** | | | | **846** | | | | **−0.18** | **[−0.31; −0.05]** | **100.0%** |

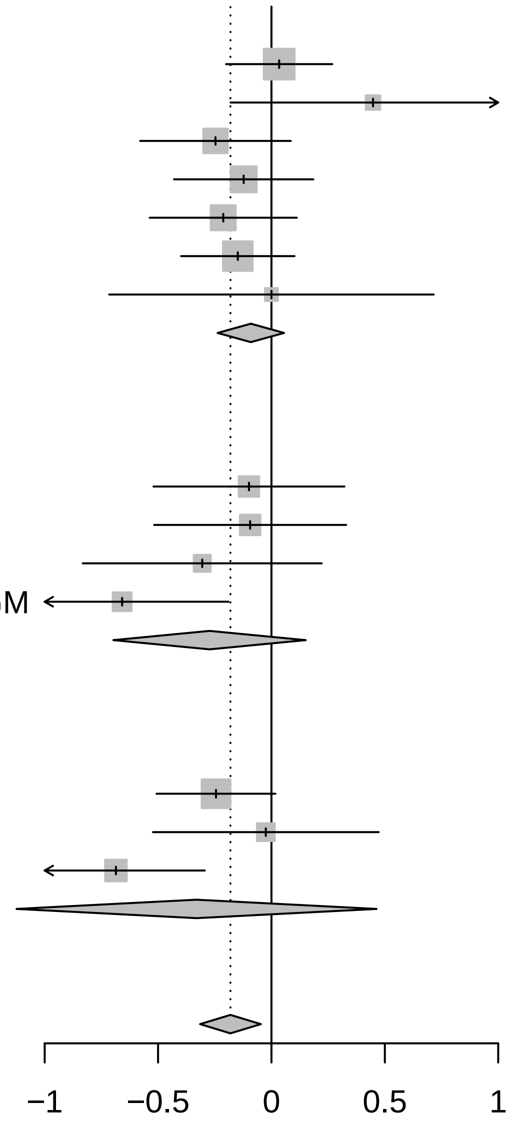


Heterogeneity: $I^2$ = 32%, $\tau^2$ = 0.0111
Test for subgroup differences: $\chi^2_2$ = 2.73, df = 2 ($p$ = 0.26)

**B-** Use of technology and HFS−B score (RCT): Change in hypoglycemia

| Study | Experimental Total | Experimental Mean | Experimental SD | Technology | Control Total | Control Mean | Control SD | Control | SMD | 95%−CI | Weight |
|---|---|---|---|---|---|---|---|---|---|---|---|
| **Less** | | | | | | | | | | | |
| Lind (2017) | 140 | 0.80 | 0.6800 | rtCGM | 140 | 0.81 | 0.6700 | CBG | −0.01 | [−0.25; 0.22] | 12.9% |
| Polonsky (2017) | 102 | 13.48 | 10.6300 | rtCGM | 53 | 17.73 | 14.9200 | CBG | −0.35 | [−0.68; −0.01] | 6.4% |
| Oskarsson (2018) | 82 | 14.90 | 11.8000 | isCGM | 81 | 18.40 | 13.5000 | CBG | −0.27 | [−0.58; 0.03] | 7.5% |
| Heinemann (2018) | 75 | 24.00 | 15.1852 | rtCGM | 66 | 26.00 | 19.2593 | CBG | −0.12 | [−0.45; 0.22] | 6.5% |
| Reddy (2018) | 20 | 30.00 | 19.6200 | rtCGM | 20 | 31.00 | 22.5900 | isCGM | −0.05 | [−0.67; 0.57] | 1.8% |
| Bosi (2019) | 75 | 18.20 | 13.2000 | SAP | 72 | 21.40 | 15.3000 | CSII | −0.22 | [−0.55; 0.10] | 6.7% |
| Pratley (2020) | 103 | 9.10 | 8.6667 | rtCGM | 100 | 10.40 | 9.6296 | CBG | −0.14 | [−0.42; 0.13] | 9.3% |
| Burckhard (2021) | 15 | 38.17 | 16.3600 | AID | 15 | 45.50 | 17.1800 | CSII | −0.43 | [−1.15; 0.30] | 1.4% |
| Visser (2021) | 124 | 15.40 | 11.9300 | rtCGM | 122 | 18.00 | 12.1200 | isCGM | −0.22 | [−0.47; 0.04] | 11.3% |
| McAuley (2022) | 15 | 4.50 | 4.0900 | AID | 15 | 4.80 | 3.2700 | SAP | −0.08 | [−0.79; 0.64] | 1.4% |
| **Random effects model** | **751** | | | | **684** | | | | **−0.17** | **[−0.25; −0.09]** | **65.2%** |
| Heterogeneity: $I^2 = 0\%$, $\tau^2 = 0$ | | | | | | | | | | | |
| **ND** | | | | | | | | | | | |
| Little (2014) (a) | 41 | 24.00 | 17.0000 | CSII | 46 | 25.00 | 17.0000 | MDI | −0.06 | [−0.48; 0.36] | 4.0% |
| Little (2014) (b) | 45 | 24.00 | 17.0000 | rtCGM | 41 | 25.00 | 17.0000 | CBG | −0.06 | [−0.48; 0.36] | 4.0% |
| Choudhary (2022) | 35 | 19.90 | 15.2600 | AID | 39 | 25.60 | 15.9800 | MDI+isCGM | −0.36 | [−0.82; 0.10] | 3.3% |
| Wheeler (2022) | 28 | 18.18 | 10.4400 | AID | 28 | 19.80 | 10.4400 | SAP | −0.15 | [−0.68; 0.37] | 2.6% |
| **Random effects model** | **149** | | | | **154** | | | | **−0.15** | **[−0.38; 0.08]** | **13.9%** |
| Heterogeneity: $I^2 = 0\%$, $\tau^2 = 0$ | | | | | | | | | | | |
| **NI** | | | | | | | | | | | |
| Beck (2010) | 120 | 25.30 | 15.8000 | rtCGM | 106 | 27.70 | 17.3000 | CBG | −0.14 | [−0.41; 0.12] | 10.4% |
| Hermanides (2011) | 35 | 24.10 | 20.2000 | SAP | 28 | 20.30 | 16.9000 | MDI | 0.20 | [−0.30; 0.70] | 2.9% |
| Kropff (2017) | 31 | 11.70 | 10.1000 | AID | 31 | 11.60 | 9.9000 | SAP | 0.01 | [−0.49; 0.51] | 2.9% |
| Kudva (2021) | 81 | 25.00 | 15.0000 | AID | 39 | 27.00 | 22.0000 | SAP | −0.11 | [−0.50; 0.27] | 4.9% |
| **Random effects model** | **267** | | | | **204** | | | | **−0.07** | **[−0.29; 0.15]** | **20.9%** |
| Heterogeneity: $I^2 = 0\%$, $\tau^2 = 0$ | | | | | | | | | | | |
| **Random effects model** | **1167** | | | | **1042** | | | | **−0.15** | **[−0.21; −0.09]** | **100.0%** |

Heterogeneity: $I^2 = 0\%$, $\tau^2 = 0$
Test for subgroup differences: $\chi^2_2 = 1.76$, df = 2 ($p$ = 0.42)

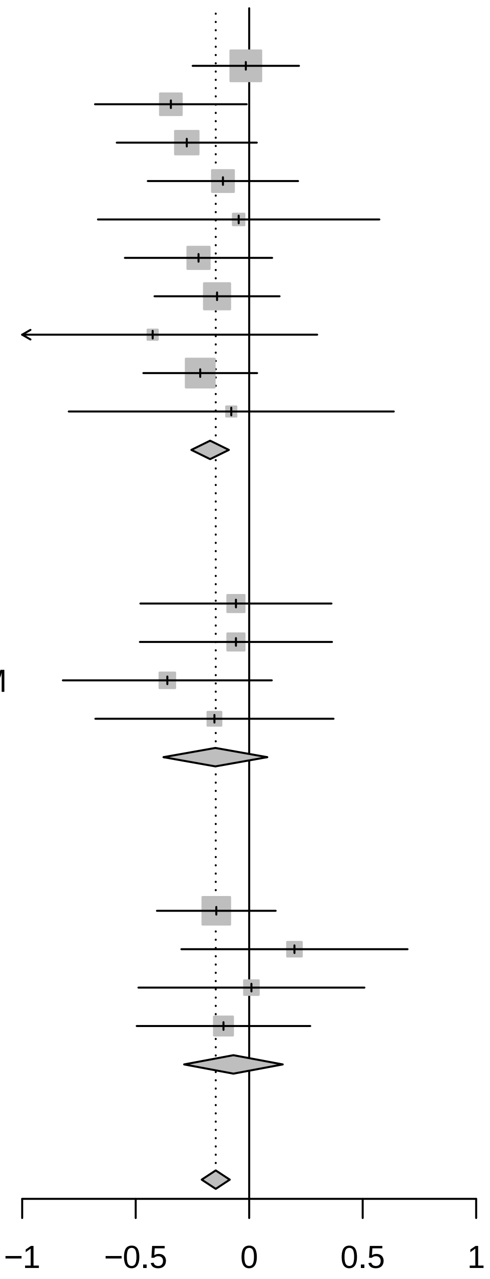


**C-** Use of technology and HFS−W score (RCT): Change in hypoglycemia

**Supplemental figure 2:** nonRCT meta-analysis results – hypoglycemia decrease subgroup analysis (Less hypoglycemia, no hypoglycemia information (NI), or no difference (ND)) (A- Total HFS score, B- HFS-Behaviour score, and C- HFS-Worry score)

| Study | Experimental Total | Experimental Mean | Experimental SD | byvar_e | Control Total | Control Mean | Control SD | control | SMD | 95%−CI | Weight |
|---|---|---|---|---|---|---|---|---|---|---|---|
| **Less** | | | | | | | | | | | |
| Boscari (2022) (a) | 38 | 28.60 | 19.4000 | rtCGM | 38 | 43.80 | 25.9000 | isCGM | −0.66 | [−1.12; −0.20] | 10.4% |
| **Nl** | | | | | | | | | | | |
| Norgaard (2013) (INTERPRET) | 179 | 28.95 | 20.1000 | SAP | 179 | 37.00 | 26.0000 | CSII | −0.35 | [−0.55; −0.14] | 50.9% |
| Perez−Garcia (2015) | 30 | 26.88 | 7.1700 | CSII | 30 | 28.04 | 7.8700 | MDI | −0.15 | [−0.66; 0.35] | 8.6% |
| Halbron (2019) | 17 | 68.00 | 17.0400 | CSII | 17 | 67.00 | 20.7400 | MDI | 0.05 | [−0.62; 0.72] | 4.9% |
| Murata (2021) | 43 | 30.30 | 13.7000 | CSII | 43 | 34.30 | 16.8000 | CSII | −0.26 | [−0.68; 0.17] | 12.3% |
| Bisio (2021) | 15 | 27.64 | 14.1100 | SAP | 15 | 24.21 | 12.4500 | SAP | 0.25 | [−0.47; 0.97] | 4.3% |
| Boscari (2022) (b) | 31 | 26.30 | 16.1000 | AID | 31 | 37.40 | 22.0000 | SAP | −0.57 | [−1.08; −0.06] | 8.6% |
| **Random effects model** | **315** | | | | **315** | | | | **−0.29** | **[−0.49; −0.08]** | **89.6%** |
| Heterogeneity: $I^2 = 0\%$, $\tau^2 = < 0.0001$ | | | | | | | | | | | |
| **Random effects model** | **353** | | | | **353** | | | | **−0.32** | **[−0.53; −0.12]** | **100.0%** |

Heterogeneity: $I^2 = 16\%$, $\tau^2 < 0.0001$

Test for subgroup differences: $\chi^2_1 = 2.23$, df = 1 ($p = 0.14$)

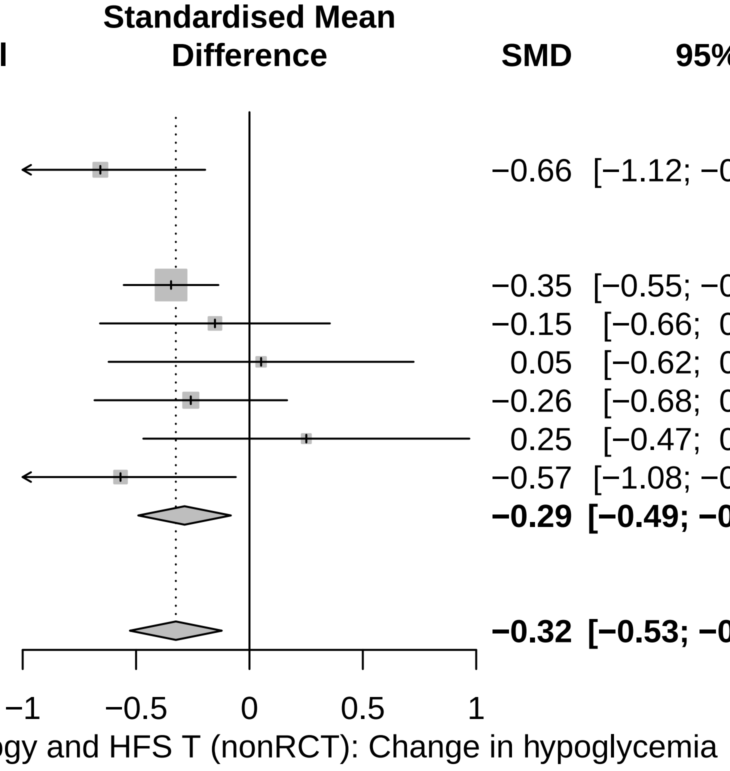

| Study | Experimental Total | Experimental Mean | Experimental SD | Technology | Control Total | Control Mean | Control SD | Control | SMD | 95%−CI | Weight |
|---|---|---|---|---|---|---|---|---|---|---|---|
| **Less** | | | | | | | | | | | |
| Rouhard (2020) | 248 | 4.40 | 3.6000 | isCGM | 248 | 5.70 | 4.1000 | CBG | −0.34 | [−0.51; −0.16] | 20.6% |
| Boscari (2022) (a) | 38 | 14.90 | 9.5000 | rtCGM | 38 | 20.80 | 10.2000 | isCGM | −0.59 | [−1.05; −0.13] | 11.4% |
| Boscari (2022) (b) | 31 | 13.00 | 8.0000 | AID | 31 | 17.50 | 9.3000 | SAP | −0.51 | [−1.02; −0.01] | 10.3% |
| **Random effects model** | **317** | | | | **317** | | | | **−0.38** | **[−0.66; −0.10]** | **42.3%** |
| Heterogeneity: $I^2 = 0\%$, $\tau^2 = 0$ | | | | | | | | | | | |
| **Nl** | | | | | | | | | | | |
| Norgaard (2013) | 179 | 13.35 | 8.9000 | SAP | 179 | 16.03 | 10.6000 | CSII | −0.27 | [−0.48; −0.07] | 19.5% |
| Shaban (2017) | 45 | 13.40 | 8.1000 | CSII | 45 | 19.10 | 9.7000 | MDI | −0.63 | [−1.06; −0.21] | 12.4% |
| Halbron (2019) | 17 | 28.00 | 2.2200 | CSII | 17 | 25.00 | 5.9260 | MDI | 0.65 | [−0.04; 1.35] | 6.9% |
| Bisio (2021) | 15 | 14.57 | 5.6900 | AID | 15 | 13.50 | 5.4300 | SAP | 0.19 | [−0.53; 0.90] | 6.5% |
| Murata (2021) | 43 | 16.90 | 6.8000 | SAP | 43 | 16.90 | 6.6000 | CSII | 0.00 | [−0.42; 0.42] | 12.4% |
| **Random effects model** | **299** | | | | **299** | | | | **−0.08** | **[−0.66; 0.49]** | **57.7%** |
| Heterogeneity: $I^2 = 67\%$, $\tau^2 = 0.1354$ | | | | | | | | | | | |
| **Random effects model** | **616** | | | | **616** | | | | **−0.26** | **[−0.57; 0.04]** | **100.0%** |

Heterogeneity: $I^2 = 56\%$, $\tau^2 = 0.0502$

Test for subgroup differences: $\chi^2_1 = 1.92$, df = 1 ($p$ = 0.17)

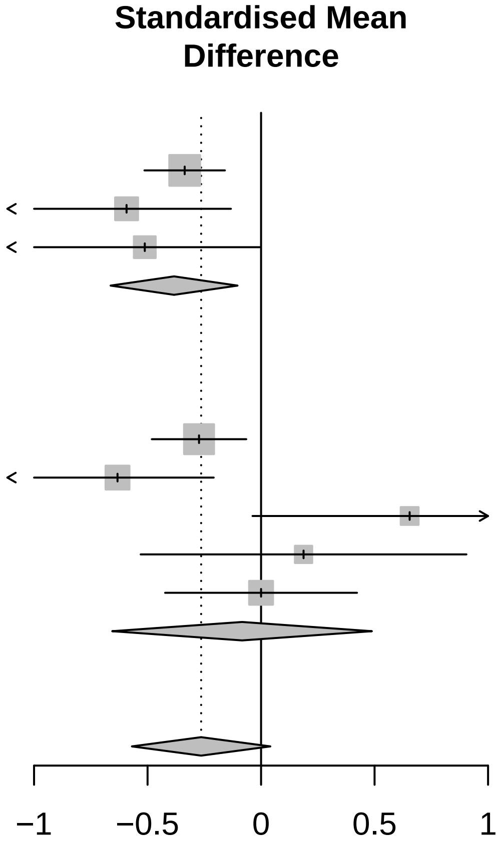


**B-** Use of technology and HFS−B score (nonRCT): Change in hypoglycemia

| Study | Experimental Total | Experimental Mean | Experimental SD | Technology | Control Total | Control Mean | Control SD | Control | SMD | 95%−CI | Weight |
|---|---|---|---|---|---|---|---|---|---|---|---|
| **Less** | | | | | | | | | | | |
| Charleer (2018) | 362 | 15.10 | 9.5000 | rtCGM | 448 | 18.60 | 10.0000 | CBG | −0.36 | [−0.50; −0.22] | 14.5% |
| Charleer (2020) | 1711 | 17.80 | 48.5400 | isCGM | 1913 | 18.10 | 54.6700 | CBG | −0.01 | [−0.07; 0.06] | 16.6% |
| Rouhard (2020) | 248 | 6.60 | 5.4000 | isCGM | 248 | 7.20 | 4.8000 | CBG | −0.12 | [−0.29; 0.06] | 13.3% |
| Wu (2020) | 15 | 22.13 | 6.8700 | AID | 15 | 26.27 | 5.8200 | SAP | −0.63 | [−1.37; 0.10] | 2.7% |
| Boscari (2022) (a) | 38 | 13.70 | 11.5000 | rtCGM | 38 | 22.90 | 16.6000 | isCGM | −0.64 | [−1.10; −0.18] | 5.6% |
| **Random effects model** | **2374** | | | | **2662** | | | | **−0.25** | **[−0.58; 0.07]** | **52.8%** |
| Heterogeneity: $I^2 = 86\%$, $\tau^2 = 0.0457$ | | | | | | | | | | | |
| **ND** | | | | | | | | | | | |
| Hermanides (2011) | 35 | 24.10 | 20.2000 | SAP | 35 | 29.80 | 19.2000 | MDI | −0.29 | [−0.76; 0.19] | 5.4% |
| **NI** | | | | | | | | | | | |
| Norgaard (2013) | 179 | 18.40 | 14.9000 | SAP | 179 | 23.39 | 15.7000 | CSII | −0.33 | [−0.53; −0.12] | 12.1% |
| Shaban (2017) | 45 | 15.00 | 12.9000 | CSII | 45 | 24.10 | 16.3000 | MDI | −0.61 | [−1.04; −0.19] | 6.3% |
| Halbron (2019) | 17 | 37.00 | 17.7800 | CSII | 17 | 38.00 | 13.3300 | MDI | −0.06 | [−0.73; 0.61] | 3.2% |
| Nefs (2019) | 35 | 15.90 | 11.0000 | rtCGM | 60 | 19.55 | 11.3900 | CBG | −0.32 | [−0.74; 0.10] | 6.3% |
| Bisio (2021) | 15 | 13.07 | 9.4300 | AID | 15 | 10.71 | 8.5900 | SAP | 0.25 | [−0.46; 0.97] | 2.8% |
| Murata (2021) | 43 | 13.50 | 8.9000 | SAP | 43 | 17.30 | 12.0000 | CSII | −0.36 | [−0.78; 0.07] | 6.2% |
| Boscari (2022) (b) | 31 | 13.20 | 9.3000 | AID | 31 | 19.80 | 14.3000 | SAP | −0.54 | [−1.05; −0.03] | 4.9% |
| **Random effects model** | **365** | | | | **390** | | | | **−0.34** | **[−0.52; −0.17]** | **41.8%** |
| Heterogeneity: $I^2 = 0\%$, $\tau^2 = < 0.0001$ | | | | | | | | | | | |
| **Random effects model** | **2774** | | | | **3087** | | | | **−0.28** | **[−0.42; −0.14]** | **100.0%** |

Heterogeneity: $I^2 = 73\%$, $\tau^2 = 0.0263$
Test for subgroup differences: $\chi^2_2 = 0.45$, df = 2 ($p$ = 0.80)

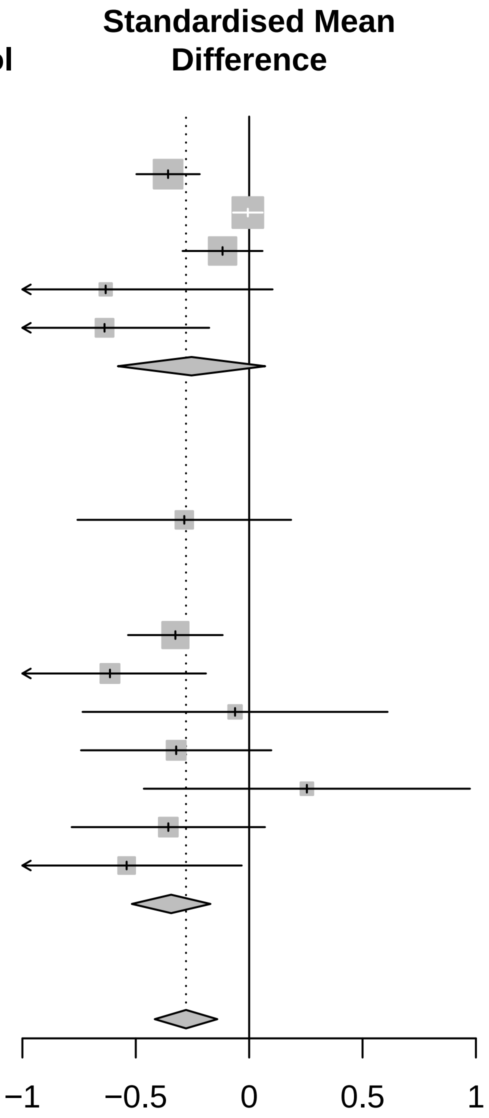


**C-** Use of technology and HFS−W score (nonRCT): Change in hypoglycemia

**Supplemental figure 3:** RCT meta-analysis results – subgroup analysis by the duration of technology use (A- HFS T score, B- HFS-Behaviour subscale, and C- HFS-Worry subscale)

| Study | Experimental Total | Experimental Mean | Experimental SD | Technology | Control Total | Control Mean | Control SD | Control | SMD | 95%−CI | Weight |
|---|---|---|---|---|---|---|---|---|---|---|---|
| **Less than 26** | | | | | | | | | | | |
| Thomas (2007) | 7 | 64.00 | 16.0000 | CSII | 7 | 67.00 | 19.0000 | MDI | −0.16 | [−1.21; 0.89] | 1.4% |
| Little (2014) (a) | 41 | 44.00 | 23.0000 | CSII | 46 | 45.00 | 25.0000 | MDI | −0.04 | [−0.46; 0.38] | 8.4% |
| Little (2014) (b) | 45 | 45.00 | 25.0000 | rtCGM | 41 | 45.00 | 24.0000 | CBG | 0.00 | [−0.42; 0.42] | 8.3% |
| Kropff (2017) | 31 | 23.50 | 16.7000 | AID | 31 | 23.50 | 16.6000 | SAP | 0.00 | [−0.50; 0.50] | 6.0% |
| Reddy (2018) | 20 | 49.50 | 34.0700 | rtCGM | 20 | 42.00 | 27.4070 | isCGM | 0.24 | [−0.38; 0.86] | 3.8% |
| Burckhard (2021) | 15 | 51.33 | 28.2200 | AID | 15 | 62.00 | 24.1300 | CSII | −0.40 | [−1.12; 0.33] | 2.8% |
| McAuley (2022) | 15 | 7.17 | 4.9000 | AID | 15 | 7.50 | 4.0900 | SAP | −0.07 | [−0.79; 0.64] | 2.9% |
| **Random effects model** | **174** | | | | **175** | | | | **−0.03** | **[−0.17; 0.11]** | **33.7%** |
| Heterogeneity: $I^2 = 0\%$, $\tau^2 = 0$ | | | | | | | | | | | |
| **26 weeks or more** | | | | | | | | | | | |
| Beck (2010) | 120 | 33.30 | 11.5000 | rtCGM | 106 | 36.00 | 13.6000 | CBG | −0.21 | [−0.48; 0.05] | 21.7% |
| Heinemann (2018) | 75 | 37.00 | 20.0000 | rtCGM | 66 | 42.20 | 25.6000 | CBG | −0.23 | [−0.56; 0.10] | 13.5% |
| Bosi (2019) | 75 | 33.70 | 19.0000 | SAP | 72 | 38.90 | 23.4000 | CSII | −0.24 | [−0.57; 0.08] | 14.1% |
| Kudva (2021) | 81 | 33.00 | 12.0000 | AID | 39 | 38.00 | 18.0000 | SAP | −0.35 | [−0.73; 0.04] | 10.1% |
| Choudhary (2022) | 35 | 35.70 | 23.3800 | AID | 39 | 47.40 | 22.8800 | MDI | −0.50 | [−0.96; −0.04] | 6.9% |
| **Random effects model** | **386** | | | | **322** | | | | **−0.27** | **[−0.40; −0.15]** | **66.3%** |
| Heterogeneity: $I^2 = 0\%$, $\tau^2 = 0$ | | | | | | | | | | | |
| **Random effects model** | **560** | | | | **497** | | | | **−0.19** | **[−0.30; −0.08]** | **100.0%** |

Heterogeneity: $I^2 = 0\%$, $\tau^2 = 0$
Test for subgroup differences: $\chi^2_1 = 10.96$, df = 1 ($p < 0.01$)

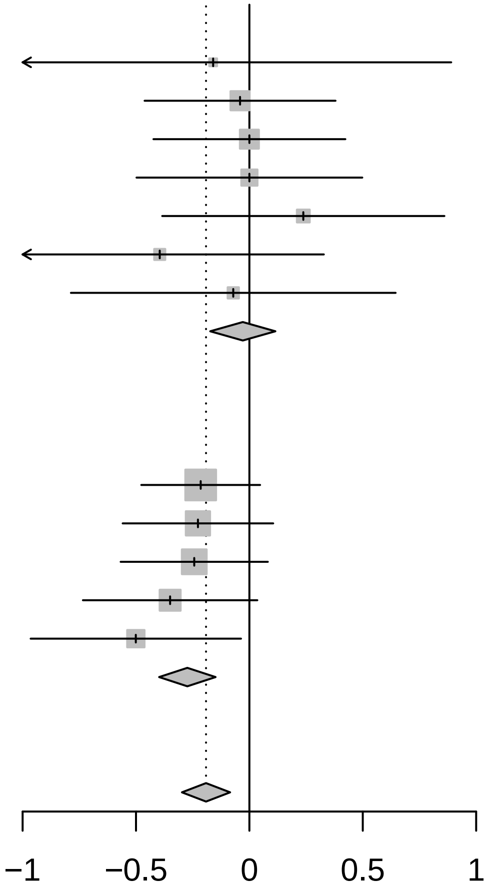


**A-** Use of technology and HFS total score (RCT): Duration of use

| Study | Experimental Total | Experimental Mean | Experimental SD | Technology | Control Total | Control Mean | Control SD | Control | SMD | 95%−CI | Weight |
|---|---|---|---|---|---|---|---|---|---|---|---|
| **26 weeks or more** | | | | | | | | | | | |
| Beck (2010) | 120 | 43.80 | 11.2000 | rtCGM | 106 | 46.80 | 13.3000 | CBG | −0.24 | [−0.51; 0.02] | 11.5% |
| Lind (2017) | 140 | 1.93 | 0.6000 | rtCGM | 140 | 1.91 | 0.5700 | CBG | 0.03 | [−0.20; 0.27] | 13.2% |
| Heinemann (2018) | 75 | 14.00 | 7.2593 | rtCGM | 66 | 16.00 | 8.8889 | CBG | −0.25 | [−0.58; 0.09] | 8.4% |
| Oskarsson (2018) | 82 | 13.40 | 5.6000 | isCGM | 81 | 14.20 | 7.3000 | CBG | −0.12 | [−0.43; 0.18] | 9.4% |
| Bosi (2019) | 75 | 15.50 | 8.7000 | SAP | 72 | 17.50 | 10.0000 | CSII | −0.21 | [−0.54; 0.11] | 8.7% |
| Visser (2021) | 124 | 19.00 | 5.4000 | rtCGM | 122 | 19.80 | 5.3500 | isCGM | −0.15 | [−0.40; 0.10] | 12.2% |
| Kudva (2021) | 81 | 43.00 | 12.0000 | AID | 39 | 52.00 | 15.0000 | SAP | −0.69 | [−1.08; −0.29] | 6.5% |
| Choudhary (2022) | 35 | 15.80 | 9.4700 | AID | 39 | 21.80 | 8.5900 | MDI+isCGM | −0.66 | [−1.13; −0.19] | 4.9% |
| **Random effects model** | **732** | | | | **665** | | | | **−0.24** | **[−0.43; −0.04]** | **74.7%** |
| Heterogeneity: $I^2 = 50\%$, $\tau^2 = 0.0227$ | | | | | | | | | | | |
| **Less than 26** | | | | | | | | | | | |
| Little (2014) (a) | 41 | 20.00 | 10.0000 | CSII | 46 | 21.00 | 10.0000 | MDI | −0.10 | [−0.52; 0.32] | 5.8% |
| Little (2014) (b) | 45 | 20.00 | 11.0000 | rtCGM | 41 | 21.00 | 10.0000 | CBG | −0.09 | [−0.52; 0.33] | 5.8% |
| Kropff (2017) | 31 | 12.20 | 8.1000 | AID | 31 | 12.40 | 7.8000 | SAP | −0.02 | [−0.52; 0.47] | 4.4% |
| Reddy (2018) | 20 | 20.00 | 11.4800 | rtCGM | 20 | 15.00 | 10.3700 | isCGM | 0.45 | [−0.18; 1.08] | 2.9% |
| Wheeler (2022) | 28 | 16.95 | 6.3000 | AID | 28 | 18.90 | 6.3000 | SAP | −0.31 | [−0.83; 0.22] | 4.0% |
| McAuley (2022) | 15 | 2.33 | 2.4500 | AID | 15 | 2.33 | 2.4500 | SAP | 0.00 | [−0.72; 0.72] | 2.3% |
| **Random effects model** | **180** | | | | **181** | | | | **−0.05** | **[−0.27; 0.18]** | **25.3%** |
| Heterogeneity: $I^2 = 0\%$, $\tau^2 = 0$ | | | | | | | | | | | |
| **Random effects model** | **912** | | | | **846** | | | | **−0.18** | **[−0.31; −0.05]** | **100.0%** |

Heterogeneity: $I^2 = 32\%$, $\tau^2 = 0.0111$
Test for subgroup differences: $\chi^2_1 = 2.48$, df = 1 ($p$ = 0.11)

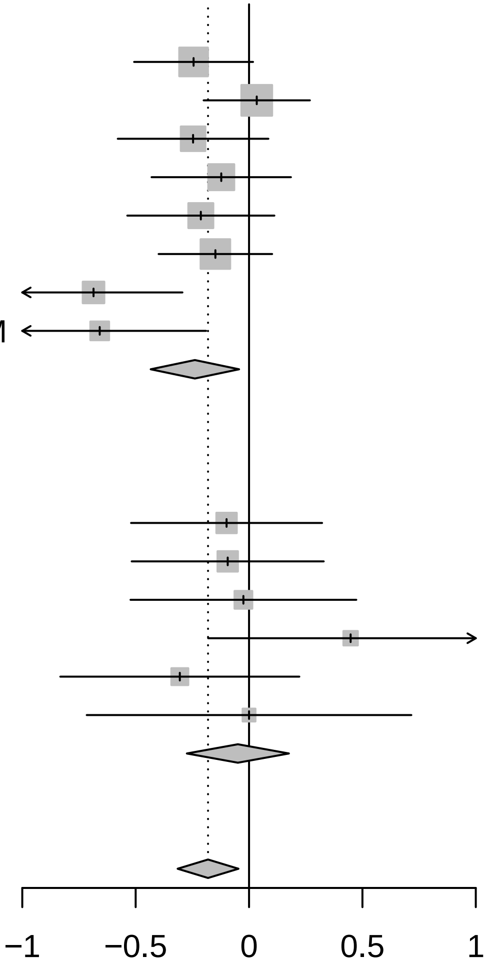


**B-** Use of technology and HFS−B score (RCT): Duration of use

| Study | Experimental Total | Experimental Mean | Experimental SD | Technology | Control Total | Control Mean | Control SD | Control | SMD | 95%−CI | Weight |
|---|---|---|---|---|---|---|---|---|---|---|---|
| **26 weeks or more** | | | | | | | | | | | |
| Beck (2010) | 120 | 25.30 | 15.8000 | rtCGM | 106 | 27.70 | 17.3000 | CBG | −0.14 | [−0.41; 0.12] | 10.4% |
| Hermanides (2011) | 35 | 24.10 | 20.2000 | SAP | 28 | 20.30 | 16.9000 | MDI | 0.20 | [−0.30; 0.70] | 2.9% |
| Lind (2017) | 140 | 0.80 | 0.6800 | rtCGM | 140 | 0.81 | 0.6700 | CBG | −0.01 | [−0.25; 0.22] | 12.9% |
| Oskarsson (2018) | 82 | 14.90 | 11.8000 | isCGM | 81 | 18.40 | 13.5000 | CBG | −0.27 | [−0.58; 0.03] | 7.5% |
| Heinemann (2018) | 75 | 24.00 | 15.1852 | rtCGM | 66 | 26.00 | 19.2593 | CBG | −0.12 | [−0.45; 0.22] | 6.5% |
| Bosi (2019) | 75 | 18.20 | 13.2000 | SAP | 72 | 21.40 | 15.3000 | CSII | −0.22 | [−0.55; 0.10] | 6.7% |
| Pratley (2020) | 103 | 9.10 | 8.6667 | rtCGM | 100 | 10.40 | 9.6296 | CBG | −0.14 | [−0.42; 0.13] | 9.3% |
| Kudva (2021) | 81 | 25.00 | 15.0000 | AID | 39 | 27.00 | 22.0000 | SAP | −0.11 | [−0.50; 0.27] | 4.9% |
| Visser (2021) | 124 | 15.40 | 11.9300 | rtCGM | 122 | 18.00 | 12.1200 | isCGM | −0.22 | [−0.47; 0.04] | 11.3% |
| Choudhary (2022) | 35 | 19.90 | 15.2600 | AID | 39 | 25.60 | 15.9800 | MDI+isCGM | −0.36 | [−0.82; 0.10] | 3.3% |
| **Random effects model** | **870** | | | | **793** | | | | **−0.14** | **[−0.23; −0.06]** | **75.7%** |
| Heterogeneity: $I^2 = 0\%$, $\tau^2 = 0$ | | | | | | | | | | | |
| **Less than 26** | | | | | | | | | | | |
| Little (2014) (a) | 41 | 24.00 | 17.0000 | CSII | 46 | 25.00 | 17.0000 | MDI | −0.06 | [−0.48; 0.36] | 4.0% |
| Little (2014) (b) | 45 | 24.00 | 17.0000 | rtCGM | 41 | 25.00 | 17.0000 | CBG | −0.06 | [−0.48; 0.36] | 4.0% |
| Kropff (2017) | 31 | 11.70 | 10.1000 | AID | 31 | 11.60 | 9.9000 | SAP | 0.01 | [−0.49; 0.51] | 2.9% |
| Polonsky (2017) | 102 | 13.48 | 10.6300 | rtCGM | 53 | 17.73 | 14.9200 | CBG | −0.35 | [−0.68; −0.01] | 6.4% |
| Reddy (2018) | 20 | 30.00 | 19.6200 | rtCGM | 20 | 31.00 | 22.5900 | isCGM | −0.05 | [−0.67; 0.57] | 1.8% |
| Burckhard (2021) | 15 | 38.17 | 16.3600 | AID | 15 | 45.50 | 17.1800 | CSII | −0.43 | [−1.15; 0.30] | 1.4% |
| McAuley (2022) | 15 | 4.50 | 4.0900 | AID | 15 | 4.80 | 3.2700 | SAP | −0.08 | [−0.79; 0.64] | 1.4% |
| Wheeler (2022) | 28 | 18.18 | 10.4400 | | 28 | 19.80 | 10.4400 | SAP | −0.15 | [−0.68; 0.37] | 2.6% |
| **Random effects model** | **297** | | | | **249** | | | | **−0.16** | **[−0.29; −0.03]** | **24.3%** |
| Heterogeneity: $I^2 = 0\%$, $\tau^2 = 0$ | | | | | | | | | | | |
| **Random effects model** | **1167** | | | | **1042** | | | | **−0.15** | **[−0.21; −0.09]** | **100.0%** |

Heterogeneity: $I^2 = 0\%$, $\tau^2 = 0$
Test for subgroup differences: $\chi^2_1 = 0.03$, df = 1 ($p = 0.87$)

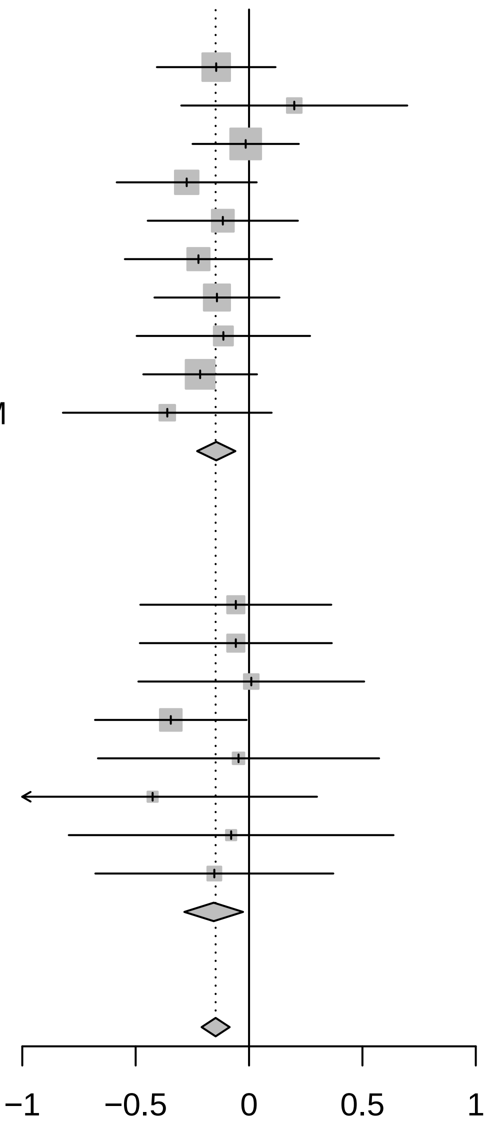


**C-** Use of technology and HFS−W score (RCT): Duration of use

**Supplemental figure 4**: nonRCT meta-analysis results – subgroup analysis by the duration of technology use (A- HFS T score, B- HFS-Behaviour subscale, and C- HFS-Worry subscale)

| Study | Experimental Total | Experimental Mean | Experimental SD | byvar_e | Control Total | Control Mean | Control SD | control | Standardised Mean Difference | SMD | 95%−CI | Weight |
|---|---|---|---|---|---|---|---|---|---|---|---|---|
| **Less than 26 weeks** | | | | | | | | | | | | |
| Boscari (2022) (b) | 31 | 26.30 | 16.1000 | AID | 31 | 37.40 | 22.0000 | SAP | | −0.57 | [−1.08; −0.06] | 8.6% |
| Boscari (2022) (a) | 38 | 28.60 | 19.4000 | rtCGM | 38 | 43.80 | 25.9000 | isCGM | | −0.66 | [−1.12; −0.20] | 10.4% |
| Bisio (2021) | 15 | 27.64 | 14.1100 | SAP | 15 | 24.21 | 12.4500 | SAP | | 0.25 | [−0.47; 0.97] | 4.3% |
| **Random effects model** | **84** | | | | **84** | | | | | **−0.39** | **[−1.55; 0.77]** | **23.3%** |
| Heterogeneity: $I^2$ = 57%, $\tau^2$ = 0.1128 | | | | | | | | | | | | |
| **26 weeks or more** | | | | | | | | | | | | |
| Perez−Garcia (2015) | 30 | 26.88 | 7.1700 | CSII | 30 | 28.04 | 7.8700 | MDI | | −0.15 | [−0.66; 0.35] | 8.6% |
| Halbron (2019) | 17 | 68.00 | 17.0400 | CSII | 17 | 67.00 | 20.7400 | MDI | | 0.05 | [−0.62; 0.72] | 4.9% |
| Murata (2021) | 43 | 30.30 | 13.7000 | CSII | 43 | 34.30 | 16.8000 | CSII | | −0.26 | [−0.68; 0.17] | 12.3% |
| Norgaard (2013) (INTERPRET) | 179 | 28.95 | 20.1000 | SAP | 179 | 37.00 | 26.0000 | CSII | | −0.35 | [−0.55; −0.14] | 50.9% |
| **Random effects model** | **269** | | | | **269** | | | | | **−0.28** | **[−0.48; −0.09]** | **76.7%** |
| Heterogeneity: $I^2$ = 0%, $\tau^2$ = 0 | | | | | | | | | | | | |
| **Random effects model** | **353** | | | | **353** | | | | | **−0.32** | **[−0.53; −0.12]** | **100.0%** |

Heterogeneity: $I^2$ = 16%, $\tau^2 < 0.0001$
Test for subgroup differences: $\chi^2_1 = 0.14$, df = 1 ($p$ = 0.71)

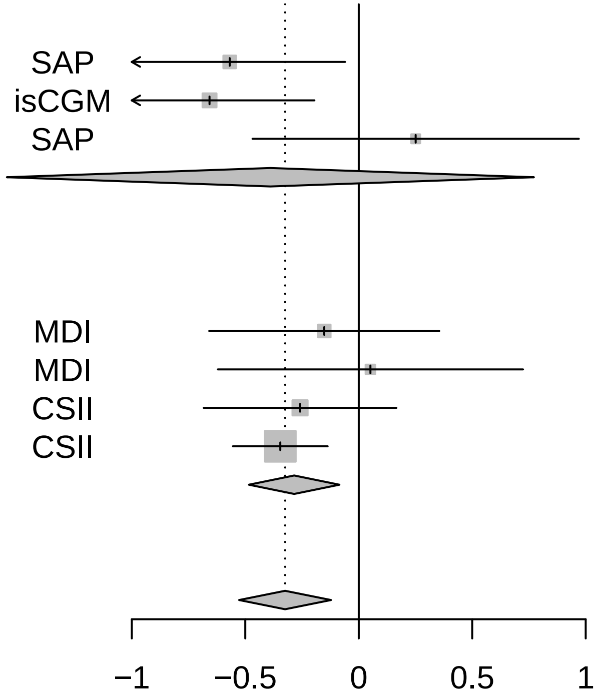


**A-** Use of technology and HFS T (nonRCT): Duration of use

| Study | Experimental Total | Experimental Mean | Experimental SD | Technology | Control Total | Control Mean | Control SD | Control | SMD | 95%−CI | Weight |
|---|---|---|---|---|---|---|---|---|---|---|---|
| **26 weeks or more** | | | | | | | | | | | |
| Norgaard (2013) | 179 | 13.35 | 8.9000 | SAP | 179 | 16.03 | 10.6000 | CSII | −0.27 | [−0.48; −0.07] | 19.5% |
| Shaban (2017) | 45 | 13.40 | 8.1000 | CSII | 45 | 19.10 | 9.7000 | MDI | −0.63 | [−1.06; −0.21] | 12.4% |
| Halbron (2019) | 17 | 28.00 | 2.2200 | CSII | 17 | 25.00 | 5.9260 | MDI | 0.65 | [−0.04; 1.35] | 6.9% |
| Murata (2021) | 43 | 16.90 | 6.8000 | SAP | 43 | 16.90 | 6.6000 | CSII | 0.00 | [−0.42; 0.42] | 12.4% |
| **Random effects model** | **284** | | | | **284** | | | | **−0.12** | **[−0.92; 0.68]** | **51.2%** |
| Heterogeneity: $I^2$ = 73%, $\tau^2$ = 0.1706 | | | | | | | | | | | |
| **Less than 26 weeks** | | | | | | | | | | | |
| Rouhard (2020) | 248 | 4.40 | 3.6000 | isCGM | 248 | 5.70 | 4.1000 | CBG | −0.34 | [−0.51; −0.16] | 20.6% |
| Bisio (2021) | 15 | 14.57 | 5.6900 | AID | 15 | 13.50 | 5.4300 | SAP | 0.19 | [−0.53; 0.90] | 6.5% |
| Boscari (2022) (a) | 38 | 14.90 | 9.5000 | rtCGM | 38 | 20.80 | 10.2000 | isCGM | −0.59 | [−1.05; −0.13] | 11.4% |
| Boscari (2022) (b) | 31 | 13.00 | 8.0000 | AID | 31 | 17.50 | 9.3000 | SAP | −0.51 | [−1.02; −0.01] | 10.3% |
| **Random effects model** | **332** | | | | **332** | | | | **−0.36** | **[−0.63; −0.08]** | **48.8%** |
| Heterogeneity: $I^2$ = 17%, $\tau^2$ = < 0.0001 | | | | | | | | | | | |
| **Random effects model** | **616** | | | | **616** | | | | **−0.26** | **[−0.57; 0.04]** | **100.0%** |
| Heterogeneity: $I^2$ = 56%, $\tau^2$ = 0.0502 | | | | | | | | | | | |
| Test for subgroup differences: $\chi^2_1$ = 0.79, df = 1 ($p$ = 0.38) | | | | | | | | | | | |

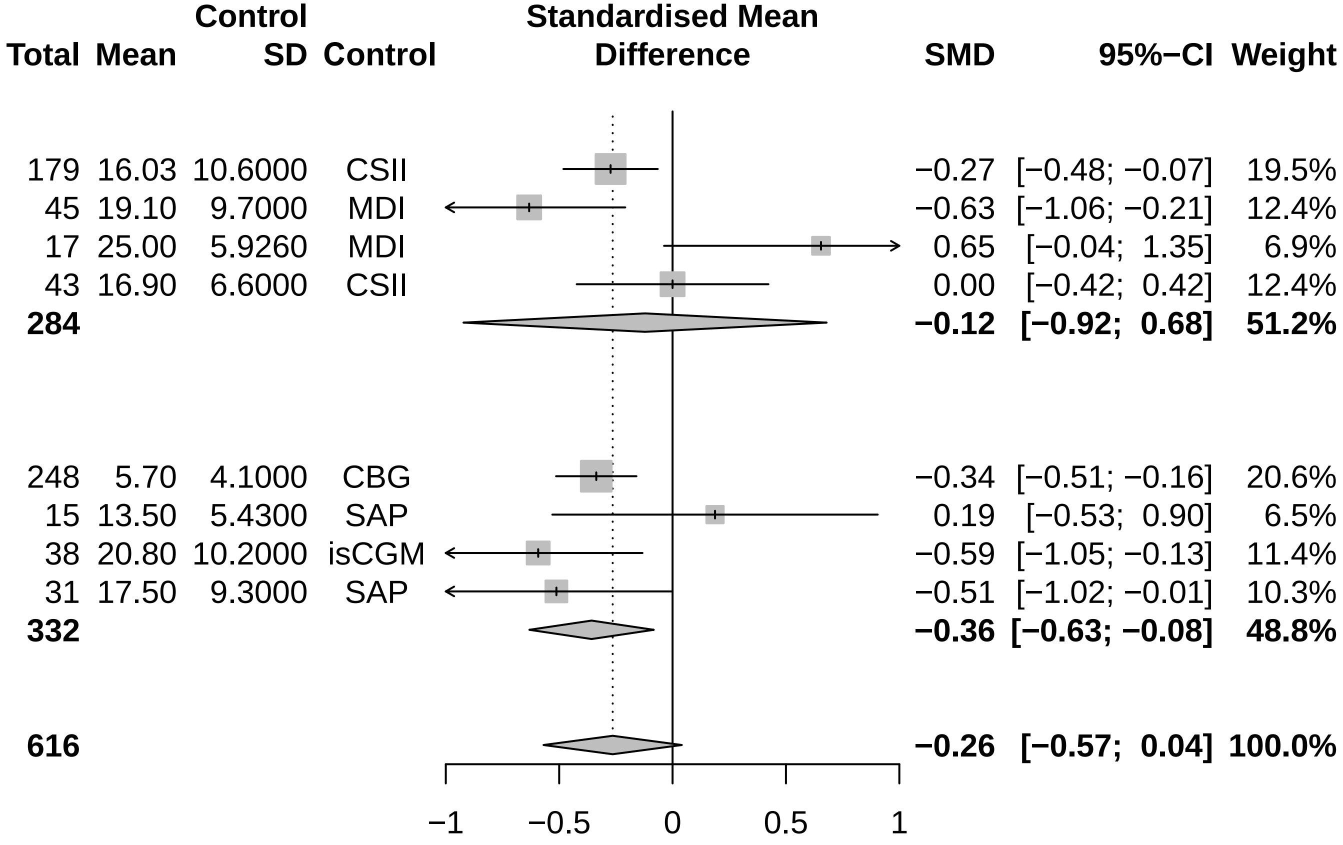


**B-** Use of technology and HFS−B score (nonRCT): Duration of use

| Study | Experimental Total | Experimental Mean | Experimental SD | Technology | Control Total | Control Mean | Control SD | Control | SMD | 95%−CI | Weight |
|---|---|---|---|---|---|---|---|---|---|---|---|
| **26 weeks or more** | | | | | | | | | | | |
| Hermanides (2011) | 35 | 24.10 | 20.2000 | SAP | 35 | 29.80 | 19.2000 | MDI | −0.29 | [−0.76; 0.19] | 5.4% |
| Norgaard (2013) | 179 | 18.40 | 14.9000 | SAP | 179 | 23.39 | 15.7000 | CSII | −0.33 | [−0.53; −0.12] | 12.1% |
| Shaban (2017) | 45 | 15.00 | 12.9000 | CSII | 45 | 24.10 | 16.3000 | MDI | −0.61 | [−1.04; −0.19] | 6.3% |
| Charleer (2018) | 362 | 15.10 | 9.5000 | rtCGM | 448 | 18.60 | 10.0000 | CBG | −0.36 | [−0.50; −0.22] | 14.5% |
| Halbron (2019) | 17 | 37.00 | 17.7800 | CSII | 17 | 38.00 | 13.3300 | MDI | −0.06 | [−0.73; 0.61] | 3.2% |
| Nefs (2019) | 35 | 15.90 | 11.0000 | rtCGM | 60 | 19.55 | 11.3900 | CBG | −0.32 | [−0.74; 0.10] | 6.3% |
| Charleer (2020) | 1711 | 17.80 | 48.5400 | isCGM | 1913 | 18.10 | 54.6700 | CBG | −0.01 | [−0.07; 0.06] | 16.6% |
| Murata (2021) | 43 | 13.50 | 8.9000 | SAP | 43 | 17.30 | 12.0000 | CSII | −0.36 | [−0.78; 0.07] | 6.2% |
| **Random effects model** | **2427** | | | | **2740** | | | | **−0.27** | **[−0.43; −0.11]** | **70.7%** |
| Heterogeneity: $I^2 = 80\%$, $\tau^2 = 0.0260$ | | | | | | | | | | | |
| **Less than 26 weeks** | | | | | | | | | | | |
| Rouhard (2020) | 248 | 6.60 | 5.4000 | isCGM | 248 | 7.20 | 4.8000 | CBG | −0.12 | [−0.29; 0.06] | 13.3% |
| Wu (2020) | 15 | 22.13 | 6.8700 | AID | 15 | 26.27 | 5.8200 | SAP | −0.63 | [−1.37; 0.10] | 2.7% |
| Bisio (2021) | 15 | 13.07 | 9.4300 | AID | 15 | 10.71 | 8.5900 | SAP | 0.25 | [−0.46; 0.97] | 2.8% |
| Boscari (2022) (a) | 38 | 13.70 | 11.5000 | rtCGM | 38 | 22.90 | 16.6000 | isCGM | −0.64 | [−1.10; −0.18] | 5.6% |
| Boscari (2022) (b) | 31 | 13.20 | 9.3000 | AID | 31 | 19.80 | 14.3000 | SAP | −0.54 | [−1.05; −0.03] | 4.9% |
| **Random effects model** | **347** | | | | **347** | | | | **−0.32** | **[−0.75; 0.11]** | **29.3%** |
| Heterogeneity: $I^2 = 55\%$, $\tau^2 = 0.0569$ | | | | | | | | | | | |
| **Random effects model** | **2774** | | | | **3087** | | | | **−0.28** | **[−0.42; −0.14]** | **100.0%** |

Heterogeneity: $I^2 = 73\%$, $\tau^2 = 0.0263$
Test for subgroup differences: $\chi^2_1 = 0.09$, df = 1 ($p$ = 0.76)

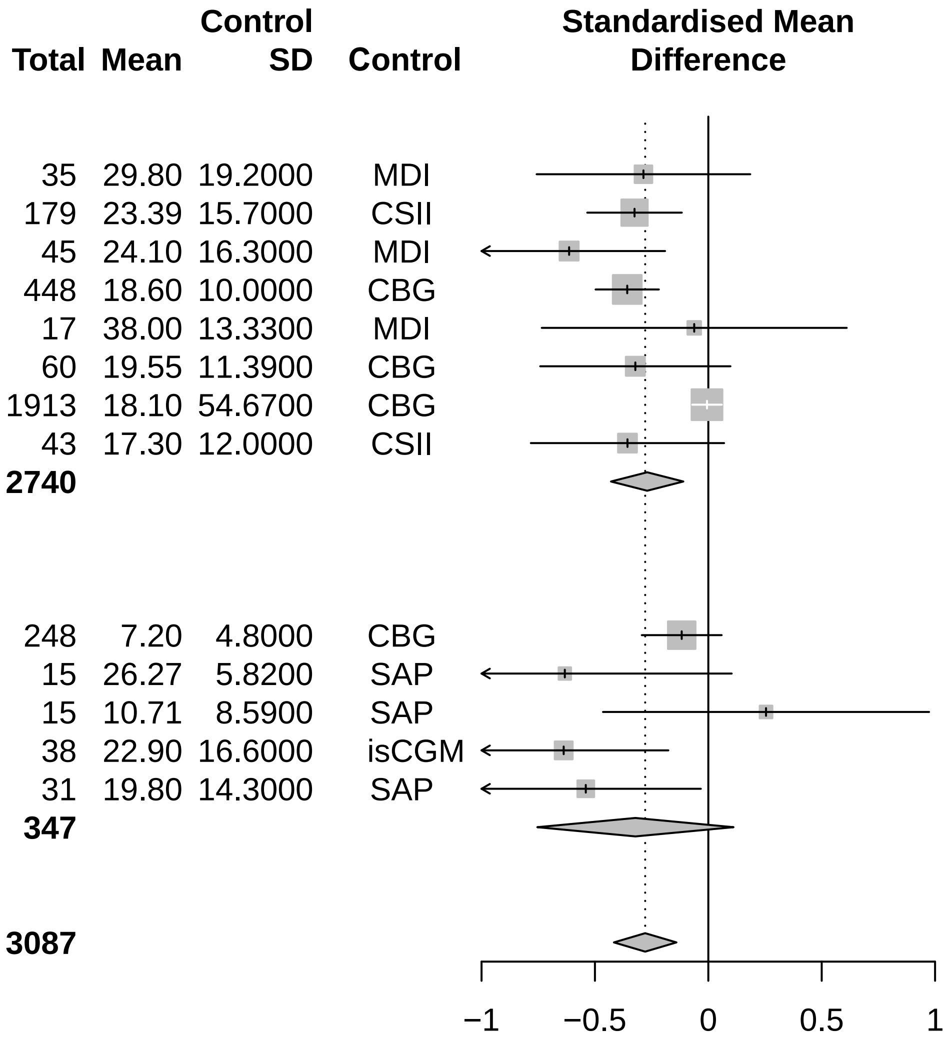


**C-** Use of technology and HFS−W score (nonRCT): Duration of use
